# Comparative genomic analysis of *Pectobacterium carotovorum* subsp. *brasiliense* SX309 provides novel insights into its genetic and phenotypic features

**DOI:** 10.1186/s12864-019-5831-x

**Published:** 2019-06-13

**Authors:** Lei Li, Lifang Yuan, Yanxia Shi, Xuewen Xie, Ali Chai, Qi Wang, Baoju Li

**Affiliations:** 1 Institute of Vegetables and Flowers, Chinese Academy of Agricultural Sciences, Beijing, 100081 China; 2 Department of Plant Pathology, College of Plant Protection, China Agricultural University, Beijing, 100193 China

**Keywords:** *Pectobacterium carotovorum* subsp. *brasiliense*, Comparative genomic analysis, Pathogenicity, Host genetic adaptation

## Abstract

**Background:**

*Pectobacterium carotovorum* subsp. *brasiliense* is a broad host range bacterial pathogen, which causes blackleg of potatoes and bacterial soft rot of vegetables worldwide. Production of plant cell wall degrading enzymes is usually critical for *Pectobacterium* infection. However, other virulence factors and the mechanisms of genetic adaptation still need to be studied in detail.

**Results:**

In this study, the complete genome of *P. carotovorum* subsp. *brasiliense* strain SX309 isolated from cucumber was compared with eight other pathogenic bacteria belonging to the *Pectobacterium* genus, which were isolated from various host plants. Genome comparison revealed that most virulence genes are highly conserved in the *Pectobacterium* strains, especially for the key virulence determinants involved in the biosynthesis of extracellular enzymes and others including the type II and III secretion systems, quorum sensing system, flagellar and chemotactic genes. Nevertheless, some variable regions of the T6SS and the CRISP-Cas immune system are unique for *P. carotovorum* subsp. *brasiliense*.

**Conclusions:**

The extensive comparative genomics analysis revealed highly conserved virulence genes in the *Pectobacterium* strains. However, several variable regions of type VI secretion system and two subtype Cas mechanism-Cas immune systems possibly contribute to the process of *Pectobacterium* infection and adaptive immunity.

**Electronic supplementary material:**

The online version of this article (10.1186/s12864-019-5831-x) contains supplementary material, which is available to authorized users.

## Background

The bacterial genus *Pectobacterium* (formerly classified as the *Erwinia* genus) is a group of facultative anaerobic, Gram-negative, non-sporulating, motile bacteria belonging to the *Pectobacteriaceae* family [1–3]. The *Pectobacterium* genus consists of heterogeneous strains, To date, thirteen *Pectobacterium* species have been described, including *P. carotovorum*, *P. atrosepticum*, *P. parmentieri*, *P. wasabiae, P. cacticida, P. betavasculorum*, *P. aroidearum, P. peruviense, P. polaris, and Candidatus* P. maceratum*, P. zantedeschiae*, *P. punjabense*, *P. aquaticum* [2, 4–7]. The *P. carotovorum* species is a complex mix of strains that show divergent characteristics. Currently, *P. carotovorum* includes four subspecies: *carotovorum*, *brasiliense*, *odoriferum*, and *actinidiae* (newly proposed but as yet not validly published) [2, 3]. Noteworthy, a number of strains previously classified as *P. carotovorum* have been separated from *P. carotovorum* recently and proposed as four new species, including *P. aroidearum*, *P. peruviense*, *P. polaris* and *Candidatus* P. maceratum [2]. To date, five complete *P. carotovorum* subspecies genome sequences have become publicly available, including *P. carotovorum* subsp. *carotovorum* PCC21 isolated from *Brassica rapa* ssp. *pekinensis* [8], *P. carotovorum* subsp. *brasiliense* BC1 isolated from *Brassica rapa* ssp. *pekinensis* [2] and *P. carotovorum* subsp. *brasiliense* BZA12 isolated from *Cucumis sativus* [9], *P. carotovorum* subsp. *odoriferum* BC S7 isolated from *Brassica rapa* ssp. *pekinensis*.

*P. carotovorum* subsp. *brasiliense* was originally reported in Brazil and has since been fully described [10]. Subsequently, *P. carotovorum* subsp. *brasiliense* has emerged as a global problem with reports from many regions of the world, including Canada, the United States, the Netherlands, Switzerland, South Africa, Kenya, South Korea, and Japan [11–13]. During 2014-2016, a devastating cucumber bacterial soft rot caused by *P. carotovorum* subsp. *brasiliense* occurred in northern China [14]. Nevertheless, very few studies have been focused on studying the complete genome of *P. carotovorum* subsp. *brasiliense*, and consequently, the pathogenicity and the genetic adaptation to the host of this subspecies remain largely unknown.

The symptoms caused by *Pectobacterium* infection include soft rot and wilts resulting from vascular invasion. Extensive studies on the *Pectobacterium* pathogens that infect vegetable crops and ornamental plants led to the identification of a number of virulence factors including extracellular degradative enzymes, diverse regulatory systems, and bacterial secretion systems, which are collectively contribute to the bacterial infections [15]. *Pectobacteria* spp. are pectinolytic pathogens, producing large quantities of plant cell wall degrading enzymes (PCWDEs). These include pectate lyase (Pel), polygalacturonase (Peh), cellulose (Cel), protease (Prt) and many others that are used to catalyze the breakdown of pectin, the primary plant cell wall component [16]. These exoenzymes are secreted via the type II secretion system (T2SS) [17] under the control of an N-acyl homoserine lactone (AHL)-dependent quorum sensing (QS) system [18]. The virulence genes related to flagella biosynthesis, bacterial colonization, and swimming motility are also regulated by the QS system in *P. carotovorum* subsp. *brasiliense* [19]. The type III secretion system (T3SS) plays an important role in the pathogenesis of most plant pathogenic bacteria [20]. However, interestingly, most the T3SS-deficient *Pectobacterium* strains exhibit similar virulence to those T3SS-encoding strains in planta [21]. The type VI secretion systems (T6SS) possibly are also important for bacterial pathogenicity and host adaptation in some bacteria, which has been largely associated with various biological functions including biofilm formation, host adaptation and bacterial survival [22].

Most archaea and many bacteria protect themselves from infection by foreign genetic elements via Cas mechanism-Cas adaptive immunity systems to ensure their survival [23]. CRISPR-Cas immunity systems evolved three stages for function: adaptation, CRISPR RNA (crRNA) biogenesis, and interference [24]. Currently, CRISPR-Cas systems include two classes, class 1 (types I, III and IV) that requires multi-Cas protein complexes for interference, and class 2 (types II, V and VI) that employs one single effector protein for interference [24]. In *E. coli*, the type I-E CRISPR-Cas interfering complex contains not only Cas1 and Cas2 but also all other components of the effector Cascade complex (*cas*A, *cas*B, *cas*C, *cas*D, *cas*E, and crRNA) and the Cas3 nuclease [25]. The subtype I-F Cas1 and Cas3 hybrid proteins interact with each other, suggesting a protein complex for adaptation and a role for the subtype I-F Cas3 proteins in both the adaptation and interference steps of the CRISPR/Cas mechanism [26]. Previous studies have shown that the CRISPR/Cas system in *P. atrosepticum* encodes six proteins including Cas1, Cas3, Csy1, Csy2, Csy3 and Csy4 [26]. Nevertheless, the biological functions of the CRISPR/Cas systems remain poorly understood in *P. carotovorum* subsp. *brasiliense*.

In this study, the complete genome sequence of *Pcb* strain SX309 that is highly virulent in a wide range of host plant species was sequenced, annotated and compared with the representative genomes of other *Pectobacterium* species and *P. carotovorum* subspecies, with a particular focus on virulence factors, regulatory mechanisms and potential genetic adaptation to the host. Through comparative genomic analysis, we found that the genes encoding PCWDEs, T2SS, T3SS, T6SS, QS system, two-component system and LPS are probably major virulence factors, and the CRISPR/Cas system may be involved in adaptive immunity. Characterization of these functional determinants among the *Pectobacterium* pathogens will provide novel insights into host-pathogen interactions.

## Methods

### Bacterial strains and genomic DNA extraction

*P. carotovorum* subsp. *brasiliense* SX309 (original number: HG1501090306) was isolated from cucumber fruit showing typical soft rot symptoms in Shanxi Province of China in February 2015 [14]. The phenotypic, biochemical characterization and host range were tested and analyzed by Meng et al [14]. This strain was typically incubated in NB (Nutrient Broth, BD, USA) liquid media at 28°C with shaking for 48 h. High-quality genomic DNA was extracted from the cultured bacteria using a QIAamp®DNA Mini Kit (Qiagen, Valencia, CA).

### Whole-genome sequencing

The complete genome sequencing of *P. carotovorum* subsp. *brasiliense* SX309 was performed at the Beijing Allwegene Technology Corporation using a Pacific Biosciences (PacBio) RS II platform with a Single Molecule Real-Time (SMRT). A SMRTbellTM template library with a 20 kb insert-size was constructed. The library was then sequenced using C4 sequencing chemistry and P6 polymerase with one SMRT cell, and the reads were trimmed on quality and length. The resulting clean reads were assembled *de novo* with the PacBio SMRT Analysis software [27] (version 2.3.0). The graphical views of genome alignments were generated using CGView software [28].

### Gene prediction and annotation

Protein coding sequences (CDS) were predicted using the NCBI Prokaryotic Genome Annotation Pipeline (PGAP, https://www.ncbi.nlm.nih.gov/genome/annotation_prok/) [29]. tRNA and rRNA genes were identified using tRNAscan-SE [30] version 2.0 and RNAmmer [31] version 1.2. The functions of the predicted proteins were annotated based on a BLASTP search against the Non-Redundant Protein Database (NR, https://blast.ncbi.nlm.nih.gov/Blast.cgi), the Pfam protein family database (http://pfam.xfam.org/), the Cluster of Orthologous Groups of proteins database (COG, https://www.ncbi.nlm.nih.gov/COG/), and the Kyoto Encyclopedia of Genes and Genomes database (KEGG, http://www.genome.jp/kegg/). Furthermore, sequence analysis was improved using the RAST analysis platform [32]. Putative signal peptides and transmembrane helices were predicted using SignalP 4.0 [33] and TMHMM 2.0 [34], respectively. The metabolic pathways were examined using a KEGG Automatic Annotation Server (KAAS, http://www.genome.jp/tools/kaas/).

### Phylogenetic analysis

Phylogenetic relationship analyses were determined from the multilocus sequence analysis (MLSA) on six housekeeping genes including 16S rRNA, *gap*A, *gyr*A, *atp*D, *rpo*A, and *rho* from twenty *Pectobacterium* spp., seven *Dickeya* spp., and eight *Erwinia* spp. GenBank accession numbers associated with the housekeeping loci of all of the strains can be found in Additional file 5: Table S3. The gene sequences were aligned using MUSCLE software and trimmed to remove ambiguously aligned regions. Subsequently, six housekeeping gene sequences were concatenated in the same order using SequenceMatrix. The phylogenetic tree was constructed using the maximum likelihoods method derived from MEGA 6.0 software [35], and 1,000 bootstrap replicates were included in a heuristic search with a random tree and the tree bisection-reconnection branch-swapping algorithm.

### Comparative analysis

According to the phylogenetic analysis, we selected eight closely related species or subspecies with released complete genomes including *P. atrosepticum* SCRI1043, *P. parmentieri* RNS08.42.1A, *P. parmentieri* SCC3193, *P. wasabiae* CFBP 3304, *P. carotovorum* subsp. *brasiliense* BC1, *P. carotovorum* subsp. *brasiliense* BZA12, *P. carotovorum* subsp. *carotovorum* PCC21, and *P. carotovorum* subsp. *odoriferum* BC S7 for genome comparison. Average nucleotide identities (ANI) values were computed for pairwise genome comparison using the OrthoANIu Algorithm (https://www.ezbiocloud.net/tools/orthoaniu) [36]. *In silico* DNA-DNA hybridization (DDH) was calculated using the Genome-to-Genome Distance Calculator (GGDC) (http://ggdc.dsmz.de/ggdc.php#) [37]. Complete genome comparisons were conducted using the progressive alignment option of the Mauve 2.3.1 comparison software [38] with the SX309 genome as the reference genome. Furthermore, synteny plots were also generated as alignments of the complete genome nucleotide sequences using MUMmer 3.22 [39]. To identify the set of common genes for the *Pectobacterium* genus and the set of genes unique to each species or subspecies, comparative analyses at the protein level were performed using an all-against-all comparison of the annotated genomes using BLASTP [40], and ortholog gene clustering analysis was implemented with the default settings [41]. Venn diagrams were created using R project language [42]. The comparative analysis of the T3SS effectors, QS system, TCS, and CRISPR/Cas system were BLASTed at the protein level using T3DB [43], SigMol [44], P2CS database [45], and CRISPRs Finder tool [46], respectively. The targets of the spacers were identified using ViroBLAST (https://indra.mullins.microbiol.washington.edu/viroblast/viroblast.php) and local BLAST analysis against NCBI plasmid genomes (ftp://ftp.ncbi.nih.gov/refseq/release/plasmid/).

### Extracellular enzyme assays

Plate assays for the activity of Pel, Peh, Cel, and Prt were conducted as described by Chatterjee et al. [17] (1995) with slight modifications. Wells were bored in the agarose medium with a No. 2 cork borer, and the bottoms were sealed with 0.8% (w/v) of molten agarose. Bacterial cells were grown in NB liquid medium overnight at 28°C and adjusted to OD_600_ = 0.8. Samples were applied to the wells, and the plates were incubated for 24 h at 28°C for Pel, Peh, and Cel and for 48 h for Prt. The Pel and Peh plates were developed with 4 N HCl, and the Cel plates were stained with 0.1% (w/v) Congo red solution for 10 min and then washed with 1 M NaCl solution three times. Haloes in the Prt plates became visible without any further treatment. Each treatment was repeated three times, and all of the experiments were repeated three times.

### Virulence assays

The virulence and symptom development caused by *P. carotovorum* subsp. *brasiliense* SX309 were assessed in cucumber plants (*Cucumis sativus*) and potato plants (*Solanum tuberosum*). Cucumber and potato stems were stab-inoculated with 10 μL of approximately 1 × 10^8^ CFU/mL bacterial suspensions of the SX309 strain. They were then incubated in a moist chamber at 28°C, and the appearance of the symptoms was periodically observed. Sterilized distilled water was used for the negative control inoculations. For each inoculation experiment, three plants were used, and the experiments were repeated three times.

### Microscopic analysis

For transmission electron microscope (TEM) observation, bacterial cells were negatively stained using 1% uranium acetate on collodion-coated 100-mesh grids. The samples were visualized using a transmission electron microscope Hitachi-7700 (Hitachi High-Technologies Corporation, Tokyo). For fluorescence electron microscope (FEM) observation, the plasmid pSMC21 containing the *gfp* gene was used to generate a GFP-tagged *P. carotovorum* subsp. *brasiliense* strain [47]. The plasmid was introduced into the bacterial cells using electroporation. The GFP-tagged SX309 strain was then visualized using a fluorescence microscope Olympus BX51. For scanning electron microscope (SEM) observation, bacterial cells in exponential and stationary phases were fixed using 2.5% glutaraldehyde. Samples were observed using a scanning electron microscope Hitachi-S3400N.

## Results

### Organism information

*P. carotovorum* subsp. *brasiliense* SX309 is a facultative anaerobic, Gram-negative, non-sporulating bacterium belonging to the *Pectobacteriaceae* family (Additional file 1: Table S1). SX309 strain is rod-shaped with a length of 1.5-2 μm and a diameter of 0.5-0.8 μm. It is motile by using peritrichous flagella (Additional file 2: Figure S1). Strain SX309 can utilize several carbon sources and grow in 5% NaCl [14]. Pathogenicity tests showed that SX309 is highly virulent in various host plants including some important vegetable crops such as cucumbers and potatoes (Additional file 3: Figure S2). Minimum information about the genome sequence (MIGS) of *P. carotovorum* subsp. *brasiliense* SX309 is summarized in Additional file 1: Table S1 [48].

### General genomic features of *P. carotovorum* subsp. *brasiliense* SX309

**Fig. 1:**
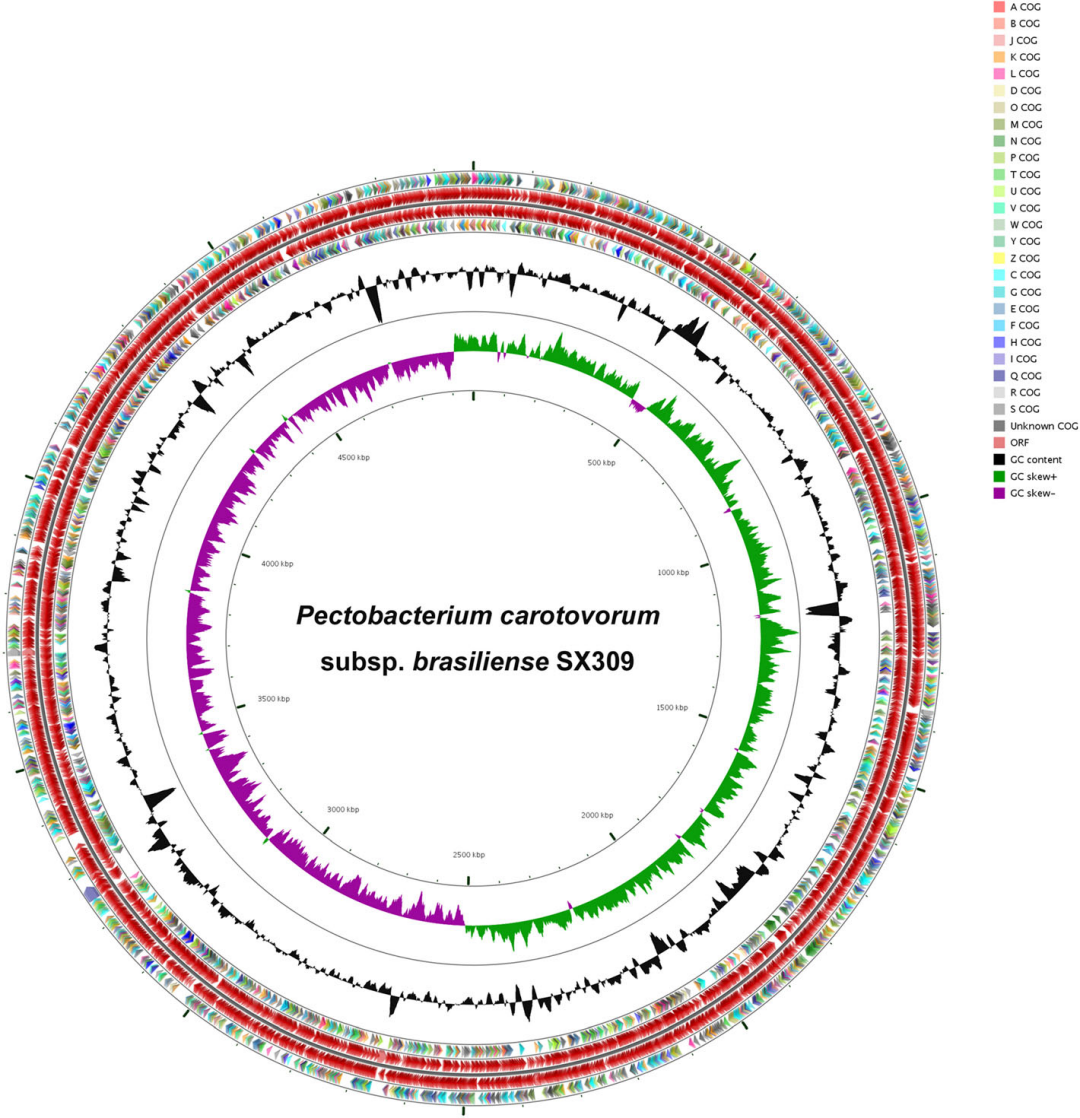
Graphical circular map of the *P. carotovorum* subsp. *brasiliense* SX309 genome performed with CGview Server. From outside to center, ring 1 and 4 show protein-coding genes oriented in the forward (colored by COG categories) and reverse (colored by COG categories) directions, respectively. Ring 2 and 3 denote genes on forward and reverse strand. Ring 5 shows G+C% content plot (black) and the inner most ring shows GC skews, where green indicates positive values and purple indicates negative values

A total of 37,555 clean reads with an average length of 12,020 bp and an N_50_ size of 16,273 bp were generated. Assembly of the clean reads resulted in a single contig with 90.89-fold coverage on average without any gap (Additional file 4: Table S2). Thus, the genome of *P. carotovorum* subsp. *brasiliense* SX309 is composed of a single circular chromosome that is 4,966,299 bp in size with no apparent autonomous plasmids (Additional file 5: Table S3 and Fig. 1). The average G+C content of the whole genome is 52.18%, which is similar to *P. carotovorum* subsp. *brasiliense* BC1 (51.8%), *P. carotovorum* subsp. *brasiliense* BAZ12 (52.00%), *P. carotovorum* subsp. *carotovorum* PCC21 (52.18%) and *P. carotovorum* subsp. *odoriferum* BC S7 (51.80%) (Table 1). In total, 4,455 open reading frames (ORFs) have been predicted in the genome of SX309. In addition to 4,252 protein coding genes (CDSs), the chromosome contains 104 RNA genes including 76 tRNA genes, 22 rRNA operons, 6 ncRNAs and 99 pseudogenes (Additional file 5: Table S3). These annotated genes are transcribed in the positive and negative directions from the perspective of the direction of DNA replication, respectively (Fig. 1). Using Pfam, SignalP, and the TMHMM database, 3,849 (86.40%), 409 (9.18%), and 19 (0.43%) of the ORFs could be classified to different groups, respectively (Additional file 5: Table S3).

**Table 1:** Genomic features of *Pectobacterium carotovorum* subsp. *brasiliense* SX309 and other *Pectobacterium* spp

Features	SX309	BC1	BAZ12	PCC21	BC S7	SCRI1043	SCC3193	CFBP3304	RNS08.42.1A
Size (bp)	4,966,299	4,920,350	4,924,809	4,842,771	4,933,575	5,064,019	5,164,411	5,043,228	5,030,841
G+C content (%)	52.18	51.80	52.00	52.18	51.80	51.00	50.40	50.60	50.40
Replicons	One chromosome	One chromosome	One chromosome	One chromosome	One chromosome	One chromosome	One chromosome	One chromosome	One chromosome
Total genes	4,455	4,472	4,476	4,340	4,340	4,615	4,821	4,579	4,564
Predicted no. of CDS	4,351	4,267	4,251	4,263	3,855	4,381	4,449	4,472	4,457
Ribosomal RNA	22	22	22	22	22	22	22	22	22
Transfer RNA	76	77	76	7	77	77	77	77	77
Other RNA	6	9	7	6	1	10	7	8	8
Pseudogene	99	97	120	121	385	125	266	184	62
GenBank sequence	CP020350.1	CP009769.1	CP024780.1	CP003776.1	CP009678.1	BX950851.1	CP003415.1	CP015750.1	CP015749.1

Functional categorization of 4,252 CDSs were analyzed using the Cluster of Orthologous Groups of proteins (COG). The results showed that 3,474 (77.98%) of the predicted genes of SX309 were assigned to the COG categories (Additional file 5: Table S3). Among these assigned genes, 42.47% are related to metabolism, 20.63% to cellular processes and signaling, and 17.13% to information storage and processing. However, 19.77% of the genes cannot be assigned in COG categories because their features and functions remain unknown (Table 2). Moreover, the RAST annotation has assigned 2,406 genes of SX309 strain into 529 subsystems. Most of the genes are associated with carbohydrates (15.83%), amino acids and derivatives (12.74%), protein metabolism (8.98%), cofactors, vitamins and pigments (7.66%), RNA metabolism (7.13%), membrane transport (5.96%), and stress response (4.29%) (Additional file 6: Figure S3).

**Table 2:** Number of genes associated with general COG functional categories

Category	Code	Value	% age	Description
Metabolism	C	208	5.08	Energy production and conversion
	E	437	10.68	Amino acid transport and metabolism
	F	82	2.00	Nucleotide transport and metabolism
	G	361	8.82	Carbohydrate transport and metabolism
	H	156	3.81	Coenzyme transport and metabolism
	I	87	2.13	Lipid transport and metabolism
	P	322	7.87	Inorganic ion transport and metabolism
	Q	85	2.08	Secondary metabolites biosynthesis, transport and catabolism
Cellular processes and signaling	D	37	0.90	Cell cycle control, Cell division, chromosome partitioning
	M	215	5.25	Cell wall/membrane biogenesis
	N	104	2.54	Cell motility
	O	139	3.40	Posttranslational modification, protein turnover, chaperones
	T	195	4.77	Signal transduction mechanisms
	U	114	2.79	Intracellular trafficking and secretion
	V	40	0.98	Defense mechanisms
	W	0	0.00	Extracellular structures
	Y	0	0.00	Nuclear structure
	Z	0	0.00	Cytoskeleton
Information storage and processing	J	180	4.40	Translation, ribosomal structure and biogenesis
	A	1	0.02	RNA processing and modification
	B	0	0.00	Chromatin structure and dynamics
	K	337	8.24	Transcription
	L	183	4.47	Replication, recombination and repair
Poorly characterized	R	479	11.71	General function prediction only
	S	330	8.06	Function unknown
	-	980	22.00	Not in COGs

### Comparison of the *P. carotovorum* subsp. *brasiliense* SX309 genome with other completely sequenced *Pectobacterium* spp.

For comparative genomic analysis of *P. carotovorum* subsp. *brasiliense* SX309, eight publicly available complete genomes of *Pectobacterium* species or subspecies including *P. carotovorum* subsp. *brasiliense* BC1(GenBank: CP009769.1 ), *P. carotovorum* subsp. *brasiliense* BZA12 (GenBank:CP024780.1), *P. carotovorum* subsp. *carotovorum* PCC21 (GenBank: CP003776.1), *P. carotovorum* subsp. *odoriferum* BC S7 (GenBank: CP009678.1), *P. atrosepticum* SCRI1043 (GenBank: BX950851.1), *P. wasabiae* CFBP 3304 (GenBank: CP015750.1), *P. parmentieri* RNS08.42.1A (GenBank: CP015749.1), and *P*. *parmentieri* SCC3193 (GenBank: CP003415.1) have been selected (Table 1). The genome size ranged from 4.84 to 5.16 Mbp, with a G+C content of 50.40-52.18% and 3855-4472 predicted CDS (Table 1). Similarly, the genomes of the seven *Pectobacterium* strains contain only one single chromosome without a plasmid.

To understand the relationships of *P. carotovorum* subsp. *brasiliense* SX309 with genome sequenced strains within the *Pectobacterium*, *Dickeya*, and *Erwinia* genera, a phylogenetic tree was constructed based on 16S rRNA and five housekeeping genes (*gap*A, *gyr*A, *atp*D, *rpo*A, *rho*) (Additional file 7: Table S4 and Additional file 8: Figure S4). As expected, the twenty *Pectobacterium* strains, seven *Dickeya* strains, and eight *Erwinia* strains were clustered into three major clades. In practice, *Pectobacterium* spp. are considered as broad-host range pathogens, except that *P. atrosepticum* has been reported almost exclusively from potatoes (*Solanum tuberosum*) and *P. betavasculorum* exclusively from sugar beets (*Beta vulgaris*). *P. carotovorum* has a broader host range and less restricted survival conditions than *P. atrosepticum*, *P. parmentieri*, and *P. wasabiae*, which are specialized to cause disease in one or few host plants only [49]. Strain SX309 is able to infect a wide range of plant species [14], which might explain the close relationship between SX309 and other *P. carotovorum* subsp. The *Dickeya* group clearly formed three distinct sub-clades. Strain EC1 was the closest homolog to *D. zeae* Ech586, followed by *D. chrysanthemi* Ech1591. In contrast, strain IPO2222 was the closest homolog to *D. dadantii* 3937, followed by *D. solani* ND14b. The *Pectobacterium* and *Dickeya* species are close relatives and were formerly classified as *Erwinia* spp. [6]. Our results of phylogenetic analysis agree with the previous findings. Thus, phylogenetic analysis based on multilocus sequences provided a strong support and an accurate classification for the species. Strain SX309 was assigned to the clade of *P. carotovorum*, which includes BC1 and BAZ12 belonging to *P. carotovorum* subsp. *brasiliense*.

The average nucleotide identity (ANI), and the genome-to-genome distance calculator, or *in silico* DDH (*is*DDH), are two of the most widely accepted bioinformatics tools that calculate whole-genome sequence similarities by comparing genomic data. A recent study showed that approximately ≥ 96% ANI values and ≥ 70% DDH values consistently grouped genomes originating from strains of the same species together [3]. In this study, we performed additional calculations on the ANI and DDH values among the representative *Pectobacterium* strains (Additional file 9: Table S5). The results showed that the ANI and DDH values between strains SX309 and BC1 were approximately 97.43% and 77.70% respectively. These findings indicated that strains SX309 and BC1 were clustered closely and occupied the same taxonomic position. Lower ANI and DDH values were obtained when BC S7, CFBP3304, RNS08.42.1A and SCC3193 were used as reference genomes.

**Fig. 2:**
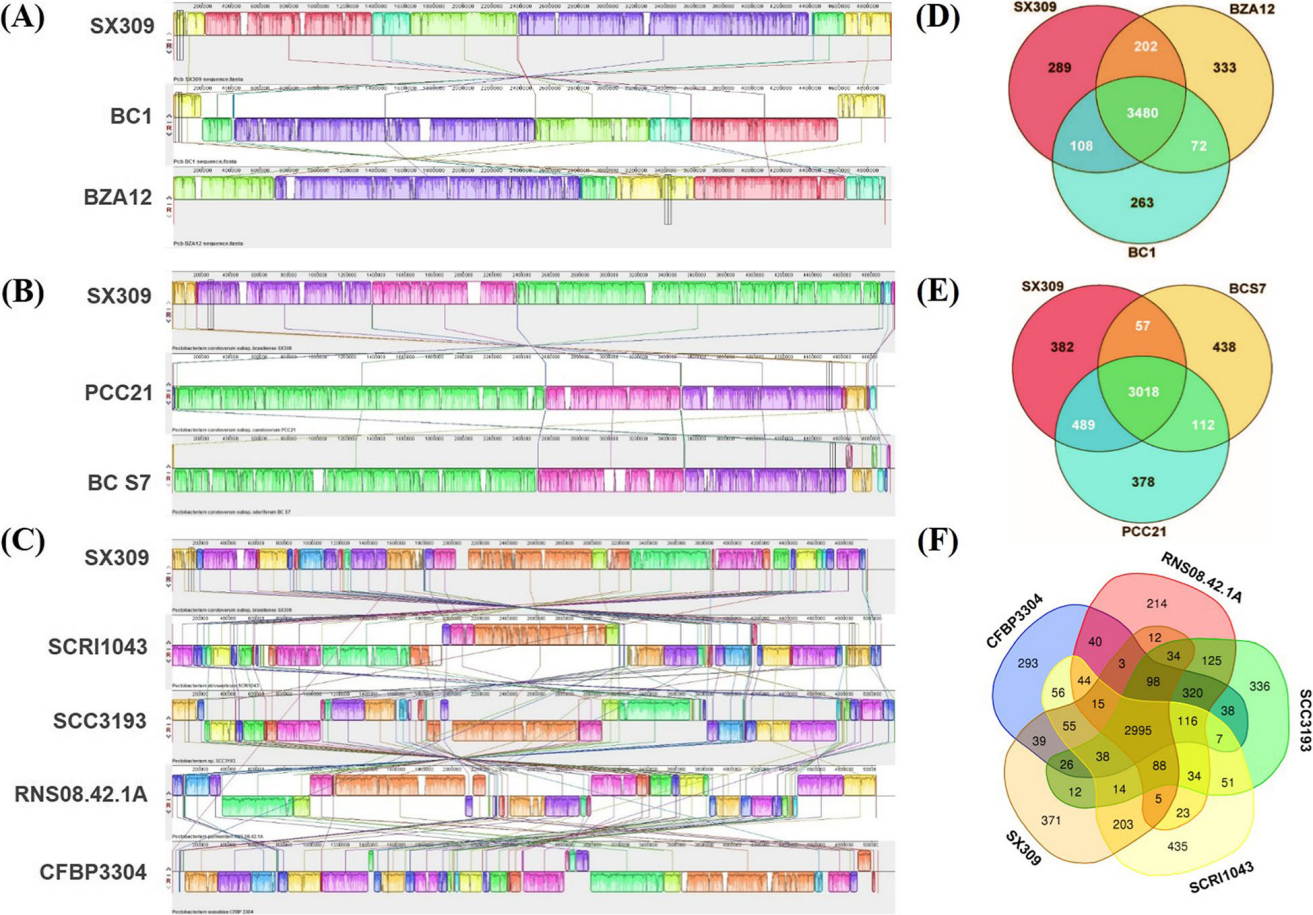
Comparison of *P. carotovorum* subsp. *brasiliense* SX309 genome sequences against other eight *Pectobacterium* genome sequences. **a** Mauve progressive alignment of SX309 genome, BC1 genome, BZA12 genome. **b** At subspecies level, mauve progressive alignment of SX309 genome, PCC21 genome, BC S7 genome. **c** At species level, mauve progressive alignment of SX309 genome, SCRI1043 genome, SCC3193 genome, CFBP 3304 genome and RNS08.42.1A genome. SX309 genome as the reference genome. Boxes with same color indicate syntenic regions. Boxes below the horizontal strain line indicate inverted regions. Rearrangements are shown by colored lines. Scale is in nucleotides. **d**, **e** and **f** Venn diagram showing the number of clusters of orthologous genes shared and unique at subspecies and species level

To evaluate the evolutionary distance among these sequenced strains within the *Pectobacterium* genus, the whole genome sequences were compared using Mauve software. At the subspecies level, the genome sequence of strain SX309 was aligned to two other *P. carotovorum* subsp. *brasiliense* (BC1 and BZA12) and its closest fully sequenced relatives, *P. carotovorum* subsp. *carotovorum* PCC21 and *P. carotovorum* subsp. *odoriferum* BC S7 (Fig. 2a and 2b). This alignment showed that the SX309 genome is much more similar to the BC1 than to the BZA12 within the *brasiliense* subsp. At the subspecies level, the SX309 genome is much more similar to the PCC21 than to the BC S7 genome, supporting the relationship described above. In comparison to PCC21, there is no significant gene insertion or deletion of large regions in *P. carotovorum* subsp. *brasiliense* SX309, but large local collinear blocks (LCB) inversion occurred. Comparison of the whole genome sequences at the species level revealed that the locations of homologous genes were different in SX309 and *P. atrosepticum* SCRI1043, *P. parmentieri* SCC3193, *P. parmentieri* RNS08.42.1A, and *P. wasabiae* CFBP 3304 (Fig. 2C). Regions with low similarity among the genome occurred frequently, and distributed randomly. Additionally, the synteny plot of the pairwise alignment supports the previous analysis that strain SX309, PCC21 and BC S7 belong to the same subspecies. Moreover, the SX309 genome is more similar to SCRI1043 than the other three *Pectobacterium* species. However, there were large numbers of changes in the LCB between SX309 and SCRI1043 during the evolution of the species (Additional file 10: Figure S5).

To identify the specific genes in *P. carotovorum* subsp. *brasiliense* SX309, we compared its genome sequence to the complete genome sequences of the eight strains that have been released (Fig.2). As shown in Fig. 2d, there were 3,480 conserved genes shared by the three strains of *P. carotovorum* subsp. *brasiliense*. SX309 shared 202 genes with BAZ12 and had 108 genes with counterparts in the BC1 genome. Furthermore, 289 unique genes were present in the genome of SX309 and the functions of most unique genes are still unknow at the moment. At the subspecies level, the core genome among SX309, PCC21 and BC S7 is composed of 3,018 orthologous genes, which represents approximately 70.98% of all the predicted genes. In addition, 382 unique genes (8.98% of the predicted genes) present in the SX309 genome were not found in the other two genomes within the same subspecies (Fig. 2e). The analysis also revealed that a core genome consisting of 2,995 genes are common to all five species, while *P. carotovorum* subsp. *brasiliense* SX309 has 371 unique genes (Fig. 2f).

### Plant cell wall-degrading enzymes

Extracellular enzyme assays showed that strain SX309 can produce pectate lyase (Pel), polygalacturonase (Peh), cellulase (Cel), and protease (Prt) (Additional file 11: Figure S6). Genome sequencing revealed the presence of the genes for the synthesis and secretion of plant cell wall-degrading enzymes in strain SX309. A total 59 known or putatively related genes encoding pectinases, cellulases and proteinases were identified in the SX309 genome. Briefly, the genome of SX309 contains 20 genes encoding pectin degradation enzymes, including *pel*N, *pel*I, *pel*A, *pel*Y, *pel*C, *pel*B, *pel*Z, *pel*W, and *pel*X for pectate lyases, *pnl* for a pectin lyase, *pem*A and *pem*B for pectinesterase, *pae*X and *pae*Y for pectin acetylesterase, *peh*X, *peh*N, *peh*A, and *peh*K for polygalacturonases, *ogl* for a oligogalacturonide lyase, and *rhi*E for a rhamnogalacturonate lyase (Additional file 12: Table S6). These pectin degradation genes were highly conserved in various *Pectobacterium* species, except that *peh*K was absent in *P. parmentieri* SCC3193 and *P. parmentieri* RNS08.42.1A. Therefore, the production of PCWDEs may be a hallmark of infection for *Pectobacterium* spp.

Similarly, 16 genes in SX309 are involved in cellulose degradation, including two endoglucanase-encoding genes *cel*V and *bcs*Z, five beta-glucosidase-encoding genes *bgl*A, *bgl*B, *bgl*D, *nag*Z, and *cel*H, and an alpha-glucosidase-encoding gene *lfa*A. These genes, associated with oligosaccharide degradation, are conserved in *Pectobacterium* stains, except that *cel*H was absent in *P. parmentieri* SCC3193 and *P. parmentieri* RNS08.42.1A (Additional file 12: Table S6). Additionally, an operon of eight genes encoding cellulose synthetase, including *bcs*C, *bcs*B, *bcs*A, *bcs*Q, *bcs*R, *bcs*E, *bcs*F, and *bcs*G, was also identified in SX309.

Moreover, 23 genes encoding proteases were detected in the SX309 genome (Additional file 12: Table S6). Among them, the six protease-encoding genes, including *prt*1, *prt*C, *prt*W, *deg*P, *deg*Q, and *glp*G encode serralysin homologs that share more than 90% similarity at the amino acid level. The four ATP-dependent Clp protease-encoding genes, including *clp*S, *clp*A, *clp*X, and *clp*P, were identified, and a lon protease encoding gene *lon* was also found in SX309.

### Secretion systems

**Fig. 3:**
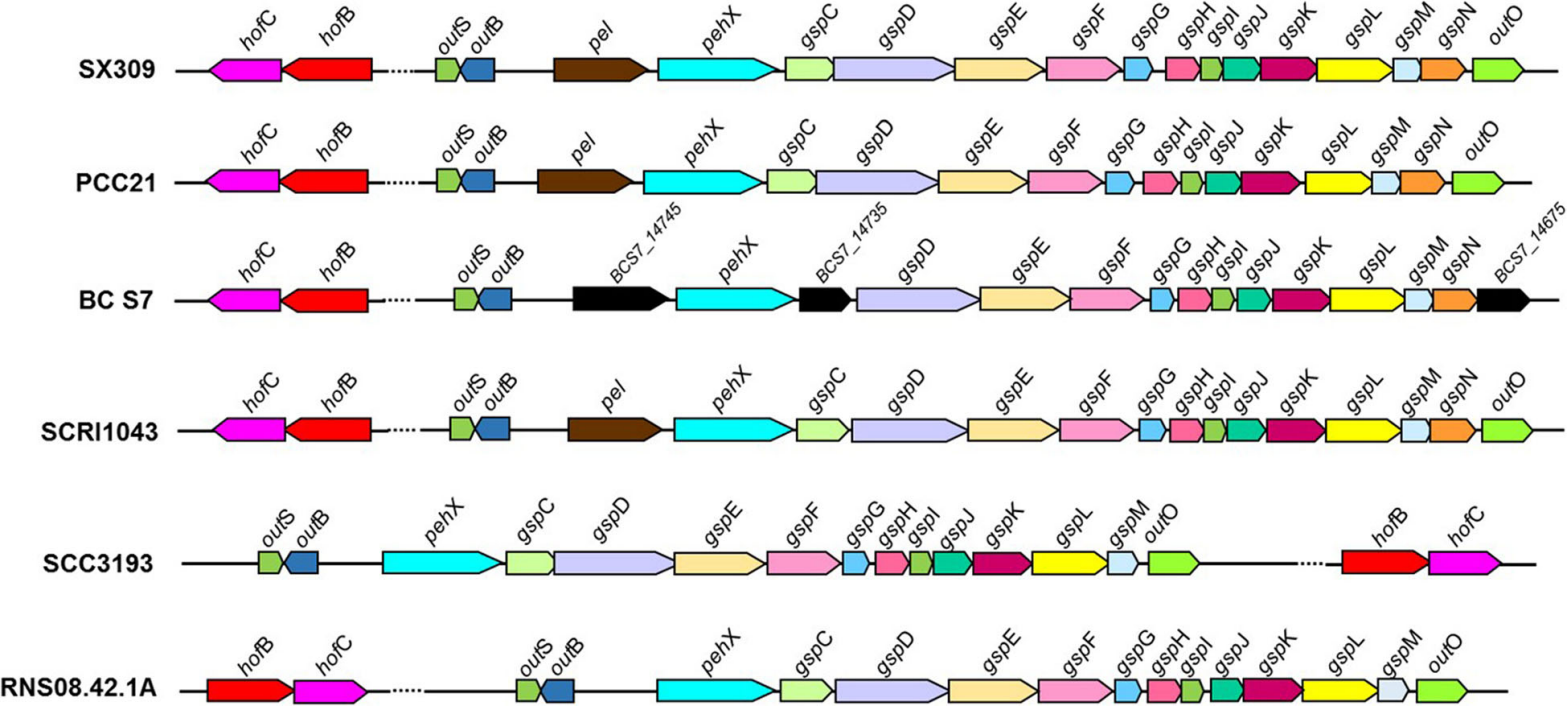
Physical map of type II secretion system in *Pectobacterium* spp. Arrows denote putative transcriptional units. The dashed line indicates long distance in the genome

The genome of SX309 contains a wide variety of secretion systems, which are closely related to bacterial pathogenicity (Additional file 13: Table S7).According to the comparative analysis, the *P. carotovorum* subsp. *brasiliense* SX309 chromosome contains a highly conserved T2SS gene cluster (*gsp*CDEFGHIJKLMN and *out*OSB) (Fig. 3), covering 17.669 kb with 15 ORFs. The *gsp* gene cluster shares an average of 90% similarity with that of various *Pectobacterium* species at the amino acid level (Additional file 13: Table S7), except that *gsp*C is absent in *P. carotovorum* subsp. *odoriferum* BC S7, and *gsp*N is absent in *P. parmentieri* SCC3193 and *P. parmentieri* RNS08.42.1A. The *out*OSB genes are also highly conserved among *Pectobacterium* spp., except that the *out*O gene is replaced by BCS7_14675 encoding a hypothetical protein in strain BC S7. Among the six *Pectobacterium* spp., the common characteristics of T2SS is that it contains *pel* and *peh*K genes upstream of *gsp*C, except the *pel* gene is absent in strains SCC3193 and RNS08.42.1A (Fig. 3). The genes involved in the secretion-signal recognition particle (Sec-SRP) system are highly conserved in all six *Pectobacterium* spp., except *sec*A and *sec*E, which are absent in strain BC S7.

Many plant pathogenic bacteria inject multiple effector proteins into plant cells via the Type III secretion system for successful infection. A large *hrp/hrc* gene cluster of 33 genes was identified in the genome of *P. carotovorum* subsp. *brasiliense* SX309. SX309 shares high similarities in the *hrp*/*hrc* gene cluster sequences with the other *Pectobacterium* species,. However, there are certain variations. For example, the *hrp/hrc* gene cluster is absent in *P. parmentieri* SCC3193 and *P. parmentieri* RNS08.42.1A but present in the other three species including *P. carotovorum* subsp. *carotovorum* PCC21, *P. carotovorum* subsp. *odoriferum* BC S7, and *P. atrosepticum* SCRI1043 (Additional file 13: Table S7). In addition, the *dsp*E and *dsp*F genes encoding the AvrE-family T3SS effectors are also conserved among *Pectobacterium* spp., except that *dsp*E is absent in *P. parmentieri* SCC3193 and *P. parmentieri* RNS08.42.1A, but *dsp*F is absent in strains BC S7, SCC3193, and RNS08.42.1A. Given that most key *hrp/hrc* genes are highly conserved in strain SX309, it is highly possible that the T3SS in SX309 could play certain roles in the bacterial pathogenicity, which awaits further investigations.

**Fig. 4:**
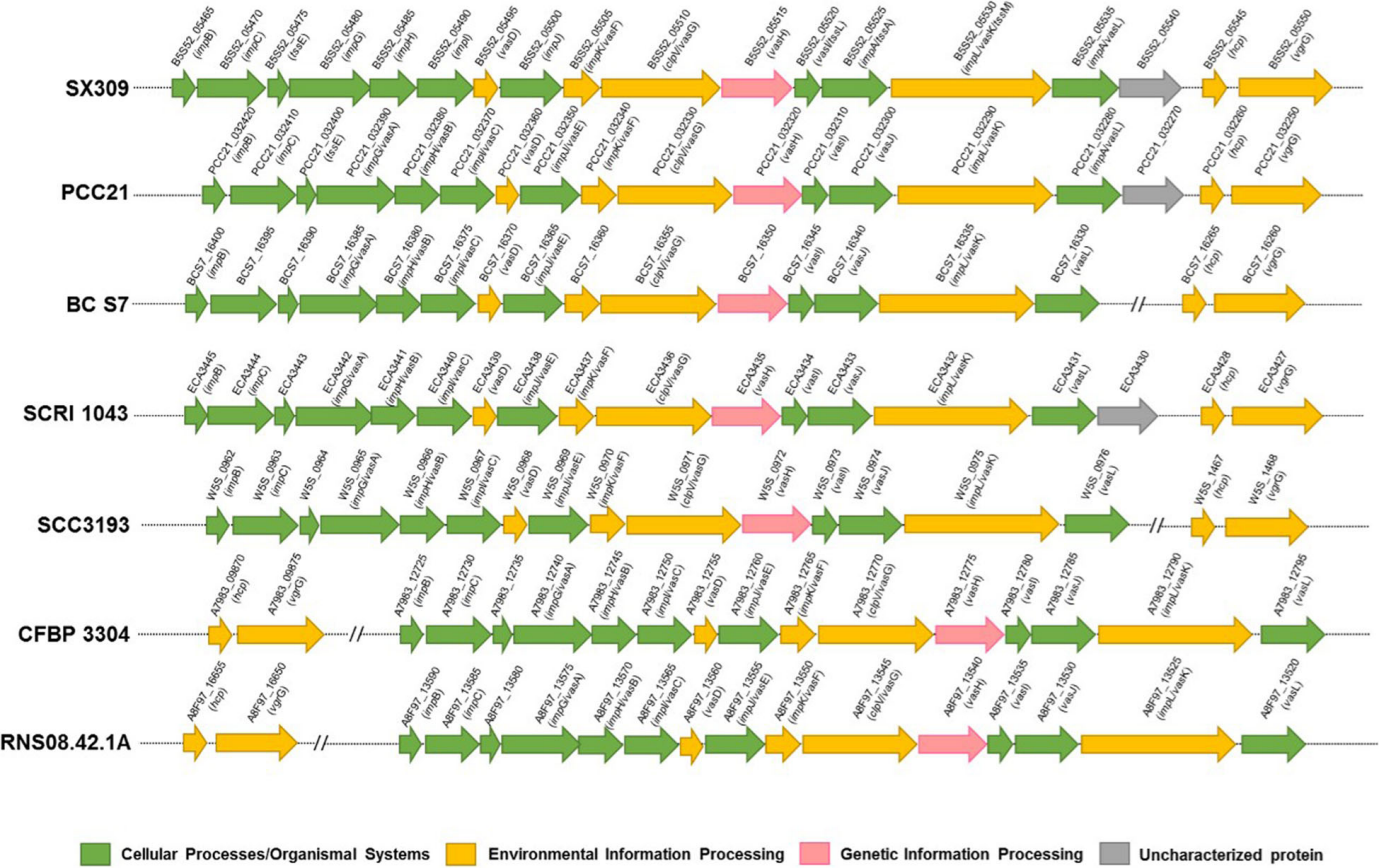
Genetic organization of the T6SS major structural gene cluster in *Pectobacterium* spp. Colored ORF indicates the genes with known function, and the same color represents the same or similar biological function. The gene encoding uncharacterized protein is indicated by gray ORF

The type VI secretion system (T6SS) is widely present in many Gram-negative bacteria, delivering toxic effector proteins into adjacent bacterial or host cells. In this study, the T6SS gene cluster of *P. carotovorum* subsp. *brasiliense* SX309 was found to have 33 genes, among which 15 were identified as core genes (Fig. 4). The 15 core T6SS genes are highly conserved in various *Pectobacterium* species and subspecies. Biological functions have been assigned for the outer membrane lipoprotein (VasD), Inner membrane proteins (ImpL and ImpK), ATPase (ClpV), and regulatory proteins or structure proteins (ImpB, ImpC, TssE, ImpG, ImpH, ImpI, ImpJ, VasH, VasI, VasJ, and VasL) [50] (Additional file 14: Table S8). In addition to the 15 core T6SS genes, there are five *vgr*G and 13 *hcp* genes that encode extracellular structural components of the secretion machine and specific effectors in SX309 genome. Nevertheless, the copy numbers of *vgr*G and *hcp* genes substantially varied among different *Pectobacterium* species and subspecies (Additional file 14: Table S8).

### Quorum-sensing systems

Quorum sensing (QS) is a cell-population density-dependent regulatory mechanism in which gene expression is coupled to the accumulation of chemical signaling molecules known as autoinducers (AI) [51]. In *P. carotovorum*, two QS systems exist that are specified by the nature of the chemical signals involved: the *N*-acyl homoserine lactones (AHLs)- and the autoinducer-2 (AI-2)- dependent signaling systems [52]. In this study, a positive reaction was observed in the AHL biosensor *Agrobacterium tumefaciens* NTL/pZLR4 (Additional file 15: Figure S7A), suggesting that SX309 could produce the AHL signals. A BLAST search of the SX309 genome revealed only one copy of *car*I (B5S52_21425) and a conserved *lux*R homolog (B5S52_21420) designated as *exp*R (Additional file 16: Table S9). The proteins encoded by *car*I/*exp*R have high sequence identity with the AHL biosynthetic and receptor proteins ExpI/ExpR of *Pectobacterium* spp. (more than 90%, respectively) at the amino acid level, except that they have low similarity with *P. parmentieri* SCC3193. In addition, *P. carotovorum* subsp. *brasiliense* SX309 has a functional *lux*S gene (B5S52_05735) and can produce an AI-2 signal (Additional file 15: Figure S7B). A BLAST search of receptors for AI-2 showed that SX309 contains one copy of *rbs*B (B5S52_21960) encoding the D-ribose ABC transporter substrate-binding protein. In the SX309 genome, two pairs of QS genes, *qse*B/*qse*C and *gac*S/*gac*A, were identified and highly conserved in SX309 and other five *Pectobacterium* strains.

### Two-component system

The genome of *P. carotovorum* subsp. *brasiliense* SX309 contains 19 TCSs (Additional file 17: Table S10). Based on the homology box, the topological characteristic of HK and the architecture of the C-terminal domain of RR [53], the 19 TCSs were grouped into five previously described subfamilies. There are nine HK/RR TCSs of the OmpR subfamily, five TCSs of the NarL subfamily, two TCSs of the CitB subfamily, two TCSs of the NtrC subfamily, and one belonging to the chemotaxis subfamily.

Sequence analysis indicated that the phoP-phoQ TCS exists in SX309 (encoded by B5S52_12350- B5S52_12355). It has a high similarity (more than 95%) at the amino acid levels with the phoP-phoQ cluster in other *Pectobacterium* strains. The genes *gac*S (B5S52_04715) and *gac*A (B5S52_08195), which encode a protein of 929 aa and 218 aa respectively, share more than 95% identity at the amino acid level with those in other *Pectobacterium* strains. Additionally, in the SX309 genome, 10 other types of putative TCSs have also been identified. They are involved in the regulation of phosphate starvation (PhoR/B), envelope stress (CpxA/R and BaeS/R), aerobic/anaerobic respiration (ArcB/A), motility (CheA/Y), capsular synthesis/virulence (RcsC/D), K^+^-limitation (KdpD/E), osmotic stress (EnvZ/OmpR), nitrogen assimilation (GlnL/G), citrate metabolism (CitA/B), and unknown function (RstB/A and BasS/R) [54] (Additional file 17: Table S10).

### Flagellar and chemotaxis genes

Two sets of genes encoding flagella biosynthetic and chemotactic proteins were found in the genome of *P. carotovorum* subsp. *brasiliense* SX309 (Additional file 18: Figure S8). The one for flagella biosynthesis is tightly clustered (B5S52_08335- B5S52_08505), and encode 39 proteins (FlhDC, FlhBAE, FlgN~K, FliR~C, FliA, and FliZ) with high protein similarity among *Pectobacterium* spp., except for the genes *fli*C and *fil*D, which showed a low similarity with those in *P. parmentieri* SCC3193 and *P. parmentieri* RNS08.42.1A (Additional file 19: Table S11). These results suggested that the entire flagellar biosynthetic region was probably acquired as a genomic island through horizontal genetic transfer in the *Pectobacterium* genus. The other set of genes for chemotaxis-related proteins are split in different clusters in the chromosome of *P. carotovorum* subsp. *brasiliense* SX309 (B5S52_08290- B5S52_08330, B5S52_00035, B5S52_06425, B5S52_08270, B5S52_10545, B5S52_12895, B5S52_14295, and B5S52_21005) (Additional file 19: Table S11). BLAST results showed that these chemotactic proteins and chemotaxis family TCSs are highly conserved (average 90% protein similarity) within the *Pectobacterium* genus.

### Lipopolysaccharide

The genes involved in the biosynthesis of LPS in SX309 were identified and clustered (Additional file 20: Table S12). Specifically, all the nine genes (*lpx*ACDHBKLM and *waa*A) and four genes (*waa*CEFQ), required for the biosynthesis of the core-lipid A complex [55], are present in the SX309 chromosome. In addition, the four genes involved in the assembly and transport of LPS in Gram-negative bacteria are also present in the SX309 genome (*lap*B and *lpt*AFG). Furthermore, the O-antigen synthetic protein encoding gene *rfb*C was also identified. Two gene clusters (*kds*ABCD and *gmh*ABCD) were also found to be highly conserved among *Pectobacterium* spp.

### Clustered regularly interspaced short palindromic repeats (CRISPR) and CRISPR-associated sequence (Cas) proteins

**Fig. 5:**
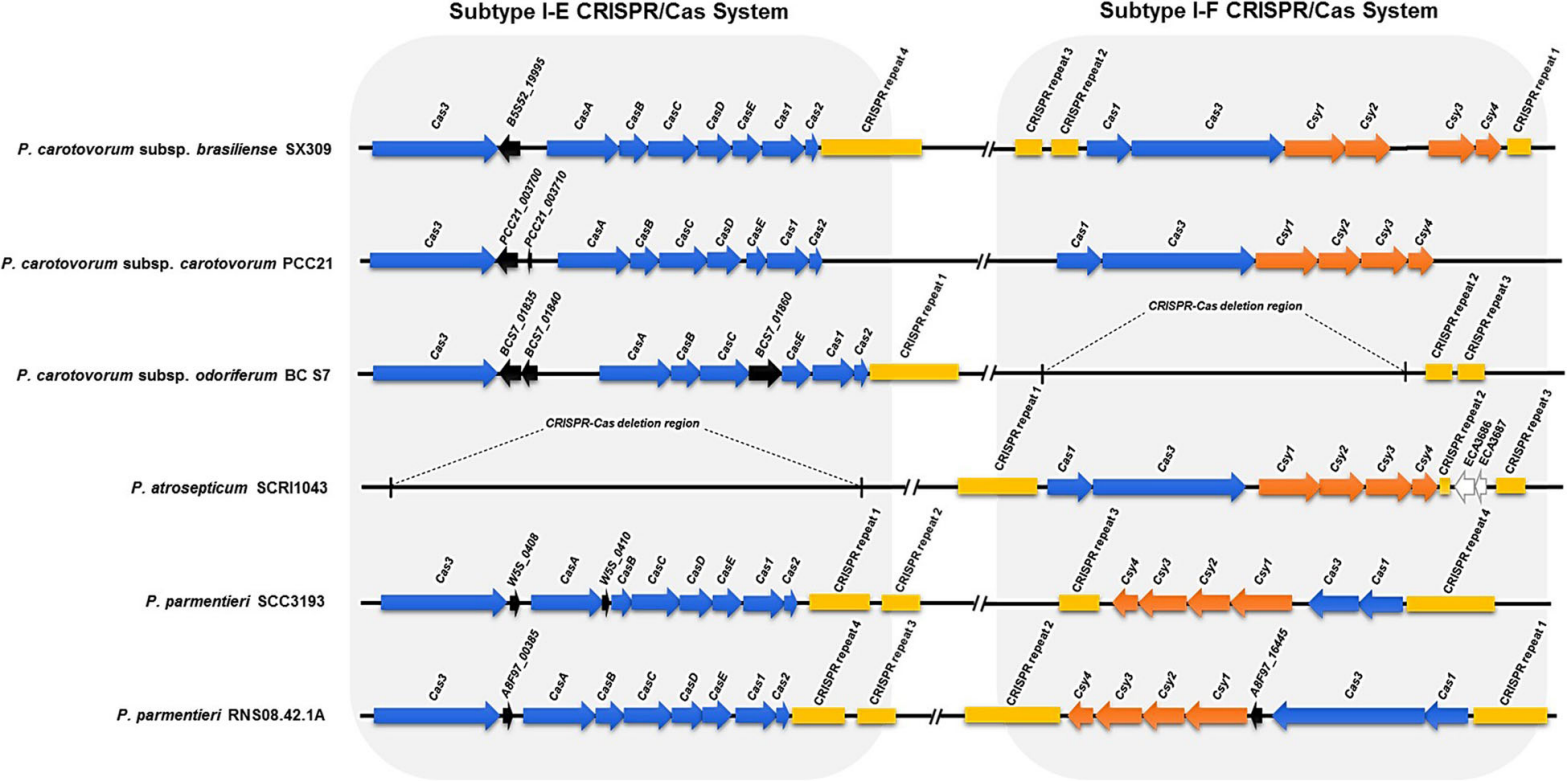
Diagram of the clustered regularly interspaced short palindromic repeats (CRISPR) with CRISPR associated proteins (Cas) system in *Pectobacterium* species. Blue indicates the subtype I-F CRISPR-associated protein, orange indicates the subtype I-E CRISPR-associated protein, yellow represents CRISPR repeats

The CRISPR-Cas systems were identified in six *Pectobacterium* genomes (Additional file 21: Table S13 and Fig. 5). *P. carotovorum* subsp. *brasiliense* SX309, *P. carotovorum* subsp. *carotovorum* PCC21, *P. parmentieri* SCC3193, and *P. parmentieri* RNS08.42.1A have two noticeable subtypes of CRISPR-Cas systems. However, *P. carotovorum* subsp. *odoriferum* BC S7 and *P. atrosepticum* SCRI1043 has only one subtype I-E CRISPR-Cas system and one subtype I-F CRISPR-Cas system respectively (Fig. 5). The subtype I-E CRISPR-Cas system in SX309 was composed of *cas*1 (B5S52_19965), *cas*2 (B5S52_19960), *cas*3 (B5S52_20000), *cas*A (B5S52_19990), *cas*B (B5S52_19985), *cas*C (B5S52_19980), *cas*D (B5S52_19975), and *cas*E (B5S52_19970). In addition, the SX309 strain subtype I-E CRISPR-Cas system contains *cas*1 (B5S52_04200), *cas*3 (B5S52_04195), *csy*1 (B5S52_04190), *csy*2 (B5S52_04185), *csy*3 (B5S52_03935), and *csy*4 (B5S52_03930) (Additional file 21: Table S13). Among the six strains, these Cas proteins are highly conserved at the amino acid level. Interestingly, the six *Pectobacterium* strains contain different numbers of CRISPR repeats (Additional file 22: Table S14 and Fig. 5). The CRISPR repeats are absent in *P. carotovorum* subsp. *carotovorum* PCC21, while other five *Pectobacterium* strains all have three or more than three CRISPR repeats with different lengths.

Based on the sequences of the CRISPR spacers, the putative CRISPR targets were also analyzed in six *Pectobacterium* strains using Viroblast or BLAST plasmid searches. The targeted sequences contained diverse phages, including those of *Pectobacterium*, *Erwinia*, and *Ralstonia*, additional bacterial phages, and various types of plasmids (Additional file 22: Table S14). In the SX309 genome, four CRISPR repeats were identified. Specifically, the CRISPR repeat sequence (CGGTTTATCCCCGCTGGCGCGGGGAACAC) conserved in *P. carotovorum* subsp. *brasiliense* SX309 and *P. parmentieri* RNS08.42.1A, contained the highest number of spacers. There were 35 spacers in *P. carotovorum* subsp. *brasiliense* SX309 and 31 spacers in *P. parmentieri* RNS08.42.1A. Three of the 35 spacers in SX309 targeted several types of phages, including *Erwinia* phage ENT90 and *Pectobacterium* phage ZF40, but did not target plasmids (Additional file 22: Table S14). Four spacers among the 31 in RNS08.42.1A targeted different types of bacteriophages, including *Pectobacterium* phage phiTE, *Pectobacterium* phage ZF40, *Erwinia* phage vB_EamM_ChrisDB, and *Erwinia* phage phiEa2809 and did not target bacterial plasmids.

## Discussion

*Pectobacterium* spp. are considered to be broad-host range pathogens, except that *P. atrosepticum* and *P. parmentieri* have been reported almost exclusively from potatoes (*Solanum tuberosum*) and *P. betavasculorum* almost exclusively from sugar beets (*Beta vulgaris*). The taxonomic position of many strains in the *Pectobacterium* genus has been re-classified in recent years [2, 9, 10]. For example, *Pcc* PC1 was classified into *P. aroidearum*, and *P. peruviense*, *P. polaris* and *Candidatus* P. maceratum were separated from *P. carotovorum.* In this study, *Pcb* SX309 was assigned to the clade of *P. carotovorum brasiliense* with the other reported *Pcb* strains (BC1 and BAZ12) based on the phylogenetic analysis. This is consistent with the findings of Meng et al [14]. Moreover, ANI and DDH values demonstrated the taxonomic position of *Pcb* SX309. The wide host range of *Pcb* SX309 also agreed with an important feature of *P. carotovorum* [3, 14].

PCWDEs including pectinases, cellulases and proteinases are key virulence factors for bacterial pathogenicity of many important plant bacterial pathogens causing soft rot disease [1]. Alignment analysis revealed that the genes related to the production of PCWDEs all exist in various *Pectobacterium* species and are highly conserved. Previous study showed that Pel and other pectinases including Peh, pectin lyase (Pnl), pectinesterase, and pectin acetylesterase play a major role in the virulence of and tissue maceration by *P. wasabiae* [56]. In our study, Pel, Peh, Cel and Pet were detected in SX309. However, the functions of these proteases in pathogenicity of *Pcb*SX309 still need to be determined.

Bacteria have evolved several sophisticated secretion systems that export a wide range of extracellular enzymes and effector proteins. In Gram-negative bacteria, these secretion systems can range from simple transporters to multi-component complexes and have been classified into six types, including type I through type VI secretion systems [57].

Many Gram-negative bacteria use the ubiquitous type II secretion system (T2SS) to translocate extracellular proteins from the periplasm across the outer membrane [58]. The T2SS is well-conserved and primarily composed of common secretion and Sec proteins, which are encoded by 12-15 general secretory pathway (Gsp) gene clusters (GspA to GspO and GspS) that are essential for the bacteria [59]. Previous studies have revealed that pectinases and cellulases are secreted by the T2SS in *Pectobacterium*, and its inactivation led to reduced pathogenicity [60]. T2SSs were also found in *D. dadantii*, the causal agent of bacterial stem and root rot of sweet potato, and *P. carotovorum* (formerly called *E. carotovorum*), which is responsible for soft rot disease in potato and other crops [61]. Moreover, the GspD- GspC T2SS played an important role in *D. dadantii* [62]. The role of T2SS in *Pcb* SX309 remains to be determined in the future*.*

T3SSs are used by many Gram-negative pathogenic bacteria to deliver virulence proteins (known as effectors) into host cells. Once inside host cells, the effectors manipulate host defenses and promote bacterial growth [63]. Unlike in many other plant bacterial pathogens, the T3SS in *P. carotovorum* subsp. *carotovorum* appears to secrete only one effector protein, DspE [64]. Therefore, *Pectobacterium* seems do not require the T3SS for pathogenicity [21]. T3SS contributes to *P. carotovorum* growth in the leaves of *Arabidopsis thaliana* [65] at the early stages of infection and contributes to the virulence of *P. atrosepticum* on *Solanum tuberosum* [66]. However, it need to be determined whether the virulence of *Pcb* partly depend on T3SS during infection of the host plant.

A promiscuous secretion system, possibly participated in bacterial pathogenicity, is the recently identified type VI secretion systems (T6SS) in diverse Gram-negative bacteria [67]. T6SS gene clusters consist of 13 core genes that are hypothesized to be minimally necessary for function and conserved genes that vary in composition between species [68]. The *vg*rG (encoding valine/glycine-repeat protein G) gene contribute to the virulence in *Acinetobacter baumannii* ATCC 19606 [69]. In *Acidovorax avenae* subsp. *avenae* strain RS-2, disruption of the genes *ppp*A, *clp*B, *icm*F, *imp*J and *imp*M caused the reduction of biofilm formation, and mutation of *ppp*A, *clp*B, *icm*F and *hcp* resulted in the reduction in motility. The vital roles of T6SS in the virulence of strain RS-2 may be partially attributed to the reductions in Hcp secretion, biofilm formation and motility. In the *Pectobacterium* genus, for the biological functions of the T6SS, researchers have not yielded a generalizable conclusion. In *Pcc* S1, *imp*G strongly influences the virulence and hypersensitive response [70]. It was demonstrated that the PCWDEs genes (*pel*A and *prt*F) and T6SS genes (*vgr*G and *hcp*3) had the same expression profiles regulated by QS. In *P. atrosepticum* SCRI1043, and the *hcp* and *vgr*G genes are induced in response to potato extracts. However, the virulence of a single gene defective mutant that was interfered in the secretion of Hcp was reported to be stronger than that of the wild-type pathogen in potato tubers [71]. A mutant with double deletions of two machinery encoding clusters spanning 16 (W5S_0962-W5S_0978) and 23 (W5S_2418-W5S_2441) genes that included the two putative T6SS encoding loci was modestly affected in its virulence in the potato tuber slice assay [56]. To date, T6SSs in many bacteria may be involved in pathogenic or symbiotic interactions with their hosts. However, more work are needed to define the function of this intriguing system in *Pcb*.

Quorum Sensing is a special type of regulation of bacterial gene expression, usually active in conditions of a high population density of bacterial cells. QS systems are widespread among the plant soft-rotting bacteria [7].Previous research showed that *Pectobacterium* spp. produces two AHL family quorum sensing signals, i.e., *N*-3-oxooctanoyl-L-homoserine lactone (3-oxo-C8-AHL) and 3-oxohexanoyl-L-homoserine lactone (3-oxo-C6-AHL), which are encoded by the *lux*I homolog *exp*I [72, 73]. The AHL signal was detected by ExpR that belongs to the LuxR family of proteins and was transduced into cellular responses. The inactivation of *exp*I resulted in the decreased production of PCWDEs and decreased virulence [19].

The second QS system, based on the production of the AI-2 signal molecules and controlled by the S-ribosylhomocysteine lyase LuxS protein, exists in a wide variety of both Gram-negative and Gram-positive bacteria and is involved in bacterial interspecies communication [74]. The LuxS/AI-2 type QS plays a strain-dependent role in virulence of different *Pectobacterium* strains. A *lux*S homolog from a *Pectobacterium* was first reported in a derivative of *P. carotovorum* subsp. *carotovorum* ATTn10 and in *P. atrosepticum* SCRI1043 [75]. Previous study revealed that there is a correlation between the AI-2 level and the production of pectinolytic enzymes. But it lacks orthologs for both known AI-2 receptors: the LuxPQ-receptor and the Lsr ABC-transporter [76]. We hypothesize that RbsB is an alternative to the AI-2 receptors in the *Pectobacterium* strain. However, the function of the *rbs*B gene still needs to be validated.

Interestingly, a new kind of autoinducer (AI-3) was discovered in *Enterohemorrhagic Escherichia coli* (EHEC). AI-3 is perceived by the sensor kinase QseC and its cognate response regulator QseB [77]. Meanwhile, it was found that *qse*C and *qse*B were both in *Pcb*SX309, Overall, the biological significance of various QS systems, especially the LuxS/AI-2 QS system in SX309 and other *Pectobacterium* species, remains to be studied further. Previous studies show that the expression of the *rsm*A/*rsm*B genes involved in the regulation of PCWDE biosynthesis is also dependent upon the global regulatory GacA/GacS system [18].

To survive, colonize and cause disease, plant-pathogenic bacteria often modulate the expression of their genes using two-component signal transduction systems (TCSs). These systems typically consist of a sensor histidine kinase (HK) and a response regulator (RR) performing a His-Asp phosphotransfer [78].It has been reported that virulence, resistance to magainin II, and the expression of pectate lyase in *D. chrysanthemi* 3937 were mediated by the response of the PhoP-PhoQ TCSs to pH and magnesium [79]. Additionally, the GacS/GacA two-component regulators are involved in the global control of virulence in *P. carotovorum* subsp. *carotovorum* [80]*.* However, the functions of these TCSs still need to be addressed.

Bacterial flagella are complex and originated very early as organelles that provide swimming and swarming motilities and play a central role in adhesion, biofilm formation, and host invasion [81]. Flagellar proteins are normally responsible for cell motility and intracellular trafficking, secretion and vesicular transport, while the chemotactic proteins are involved in cell motility and signal transduction [82].In *D. dadantii* 3937, the mutation of *fli*A encoding a sigma factor eliminated the bacterial motility, and significantly reduced Pel production and the bacterial attachment to plant tissues [82]. Similarly, the inactivation of *flg*A, *fli*A, and *flh*B gene abolished the bacterial motility and significantly reduced the bacterial virulence in *P. carotovorum* subsp. *carotovorum* PCC21 [83]. We have observed that *Pectobacterium* cells are motile in diseased plant tissues (data not published), but whether the production of PCWDEs and secretion systems that contribute to virulence is coordinated with motility is still unclear. Thus, the functions of flagellar and chemotactic genes in *Pectobacterium* pathogenicity, especially in pathogen-host plant interactions, remain to be explored.

LPSs were shown to have complex and differing roles depending on their origin and the challenged plant. Previous research reported that different defense response patterns could be induced by the LPS of *P. atrosepticum* and *Pseudomonas corrugata* in three *Solanaceae* species, including tobacco, tomato, and potato [84]. Additionally, different signaling pathways could also be activated by LPS in *Arabidopsis thaliana* cells [85].A previous study showed that LPS are crucial for the optimal growth, survival and virulence of *P. atrosepticum* [86], but the roles of LPS in the SX309 strain remain to be determined.

The CRISPR-Cas system mediate immunity to invading genetic elements such as bacteriophages, viruses and plasmids [87]. Based on the presence of the Cas3, Cas9, and Cas10 proteins, different CRISPR-Cas systems were classified into three major types, type I, II, and III. The major types comprise further subtypes (e.g., I-A to I-F), each is characterized by a specific set of proteins [90]. Cas1 is the protein hallmark of CRISPR-mediated immunity, and Cas 1 and Cas2 were found in all CRISPR-containing organisms [23].

The key factors of the CRISPR-mediated immunity system are small CRISPR RNAs that guide nucleases to complementary target nucleic acids of invading genetic material, generally followed by the degradation of the invader [88, 89]. Previous studies revealed that the *P. atrosepticum* SCRI1043 CRISPR-Cas system contains six proteins, including Cas1, Cas3, and the four subtype I-F specific proteins Csy1, Csy2, Csy3, and Csy4, and three CRISPR repeats [26]. In *P. atrosepticum*, the Csy4 protein was identified to be responsible for processing the CRISPR RNAs into crRNAs and appears to interact with itself in the absence of other Cas proteins [90]. In our study, we found *Pcc*, *Pco*, and *Pcb* all harbor two subtypes of CRISPR/CAS system (Type I-E, I-F). In *Escherichia coli,* primed adaptation by type I-E CRISPR-Cas system occurs after the Cascade-crRNA complex interacts with a fully matching protospacer that is subject to interference [25]. However, there are relatively few reports concerning the CRISPR-Cas system in *Pectobacterium* species.

T6SS can be deployed as versatile weapons to compete with other bacterial cells or attack simple or higher eukaryotic cells and likely plays an important role in mediating a pathogenic or a symbiotic relationship between bacteria and eukaryotes in various environmental niches [68, 91–94]. Antibacterial effector toxins secreted by T6SSs contributed to the antibacterial functions, which could be neutralized by corresponding antagonistic immunity proteins to preventing self-killing or sibling-intoxication. In *Vibrio cholerae*, VgrG-3 was found to degrade peptidoglycan and hydrolyse the cell wall of Gram-negative bacteria, and the TsaB (type six secretion antitoxin B) was identified as the immunity protein. In *Dickeya dadantii* 3937, Rhs played an important role in intercellular competition, which is linked with the VgrG component of T6SS [94]. The functions of T6SSs should be determined in future research.

## Conclusion

This study provided a comprehensive analysis of the complete genome of *P. carotovorum* subsp. *brasiliense* strain SX309, a causative agent of bacterial soft rot disease. The genomic analysis of strain SX309 has shown that this bacterium belongs to the *P. carotovorum* subsp. *brasiliense*. The chromosome organization and structure in *P. carotovorum* subsp. *brasiliense* strain SX309 is most similar to that of *P. carotovorum* subsp. *brasiliense* BC1, which is consistent with the finding that SX309 and BC1 are closely related based on multilocus sequence analysis. To our knowledge, the primary pathogenicity determinants of *Pectobacterium* are the coordinated production of PCWDEs that macerate host tissue and release nutrients for bacterial growth. These extracellular enzymes are secreted by the T2SS under the control of QS system [17]. Type III secretion system (T3SS) genes are not involved in the secretion of exoenzymes, but they still play a crucial role in *Pectobacterium* pathogenicity [21]. Compared to other pathogenic bacteria belonging to the *Pectobacterium* genus, the genome of *P. carotovorum* subsp. *brasiliense* SX309 encodes many similar virulence factors, including the PCWDE biosynthetic genes, T2SS and T3SS genes, bacterial QS genes, flagella and chemotactic genes. Moreover, comparative analysis revealed that the *Pectobacterium* strains harbor the type VI secretion system and CRISPR-Cas immune system genes, which were suggested to contribute to bacterial virulence and adaptive immunity. However, the functions of these genes remain to be elucidated in *P. carotovorum* subsp. *brasiliense*.

In summary, the comprehensive analyses of the genomes of *Pectobacterium* strains provide new insights for the conservation and evolution processes of virulence elements in these important bacterial pathogens. Knowledge of the variability and specificities of the *Pectobacterium* organisms could contribute to a better understanding of the molecular mechanisms of unique genetic features and pathogenesis.

## Additional files

Additional file 1: **Table S1.** Classification and general features of *Pectobacterium carotovorum* subsp. *brasiliense* SX309 according to the MIGS recommendations. (DOCX 15 kb)

Additional file 2: **Figure S1.** General characteristics of *P. carotovorum* subsp. *brasiliense* SX309. Image of SX309 cells using transmission electron microscopy (A) and fluorescent microscopy (B). Image of SX309 cells from the exponential growth phase (C) and the stationary phase (D), respectively. (TIF 2925 kb)

Additional file 3: **Figure S2.** *P. carotovorum* subsp. *brasiliense* SX309 symptoms on representative cucumber (A, *Cucumis sativus*) and potato (B, *Solanum tuberosum*) stems. The bacterial cells were used to inoculate cucumber or potato stem at 10^8^ cfu·mL^-1^. At 24 hours after inoculation, the soft rot (A) or blackleg (B) symptom were observed and photographed. (TIF 3173 kb)

Additional file 4: **Table S2.** Project information. (DOCX 14 kb)

Additional file 5: **Table S3.** Genome statistics. (DOCX 12 kb)

Additional file 6: **Figure S3.** Annotation of *P. carotovorum* subsp. *brasiliense* SX309 as generated by the Rapid Annotation using Subsystem Technology (RAST, http://rast.nmpdr.org/) webserver. (TIF 2271 kb)

Additional file 7: **Table S4.** The locus tag information of 16S rRNA genes and five housekeeping genes used for phylogenetic analysis in this study. (DOCX 19 kb)

Additional file 8: **Figure S4.** Phylogenetic tree highlighting the relative position of *P. carotovorum* subsp. *brasiliense* SX309 within other *Pectobacterium, Dickeya,* and *Erwinia* species. The phylogenetic tree was constructed based on six housekeeping genes (16S rRNA, g*ap*A, g*yr*A, *atp*D, *rpo*A, *rho*) according to the aligned gene sequences using maximum likelihoods derived from MEGA 6.0 software. Bootstrap values (1,000 replicates) are shown at the branch points. The scale bar indicates 0.02 nucleotide substitution per nucleotide position. The original hosts of bacteria were shown in the brackets. GenBank accession numbers associated to the housekeeping loci of all strains can be found in Additional file 3: Table S4. (TIF 4868 kb)

Additional file 9: **Table S5.** Percentage of average nucleotide identities (ANI)^a^ and *in silico* DNA-DNA hybridization (DDH)^b^ among the selected *Pectobacterium* genomes (XLSX 11 kb)

Additional file 10: **Figure S5.** Dot-plot analysis of linear genomic organization between *P. carotovorum* subsp. *brasiliense* SX309 and other six previously fully sequenced *Pectobacterium* genomes. The X-axis represents the SX309 genome, Y-axis represents PCC21 genome (A), BC S7 genome (B), SCRI1043 genome (C), SCC3193 genome (D), RNS08.42.1A genome (E), and CFBP 3304 genome (F), respectively. Red indicates the alignment sequence in the forward direction, blue indicates the alignment sequence in the reverse direction. (TIF 3562 kb)

Additional file 11: **Figure S6.** Production of extracellular enzymes in *P. carotovorum* subsp. *brasiliense* SX309. Plate assays for the activity of pectate lyase (Pel), polygalacturonase (Peh), cellulase (Cel), and protease (Prt). In the center point of plate, wells were made in agarose media with a no. 2 cork borer and the bottoms were sealed with 0.8% (w/v) molten agarose. Bacterial cells were grown until early stationary phase at 28°C in NB medium (noninduced). After adjustment of the optical density of cell suspensions at 600 nm to 0.6 by adding sterilized distilled water, 10 μl of the cultures were applied to each well. After incubation at 28°C, each plate was treated as described in Methods. Three independent experiments had similar results. (TIF 2528 kb)

Additional file 12: **Table S6.** Homologs of cell wall-degrading enzyme genes in *P. carotovorum* subsp. *brasiliense* SX309 and other *Pectobacterium* spp. (DOCX 23 kb)

Additional file 13: **Table S7.** Identification of homologs of type II, III and Sec-SRP secretion system genes in *P. carotovorum* subsp. *brasiliense* SX309 and other *Pectobacterium* spp. (DOCX 25 kb)

Additional file 14: **Table S8.** Genetic elements of T6SS-encoding gene clusters in pathogenic *Pectobacterium* spp. were summarized and the presence of the key T6SS structure genes is indicated for the analysed genomes. (DOCX 18 kb)

Additional file 15: **Figure S7.** The detection of AI-1 and AI-2 QS signal biosynthesis in *P. carotovorum* subsp. *brasiliense* SX309. (A) Analysis of *N*-acyl-homoserine lactone (AHL) produced by *P. carotovorum* subsp. *brasiliense* SX309. β-Galactosidase activity of the *tra*G*-lac*Z fusion in the biosensor strain *A. tumefaciens* NTL4 (pZLR4) was measured after incubation with AHL extracted from the wild-type SX309. All experiments were performed in triplicate, and error bars indicate standard deviation; those with a different letter are significantly different according to least signification difference test (*P*<0.05). (B) Induction of bioluminescence in *Vibrio harveyi* reporter strain BB170 by cell-free medium (CFM) from *P. carotovorum* subsp. *brasiliense* SX309. **S**terile AB medium and CFM from 5 mL cultures of *V. harveyi* BB120 were used as negative and positive controls. The baseline is the value when uninoculated (sterile) CFM alone at 2, 4, 6 h were used. Each bar represents the mean (±SD) of triplicate experiments. (TIF 1825 kb)

Additional file 16: **Table S9.** Identification of homologs of quorum sensing genes in *P. carotovorum* subsp. *brasiliense* SX309 and other *Pectobacterium* spp. (DOCX 16 kb)

Additional file 17: **Table S10.** Homolog of two-component system encoding genes in *P*. *carotovorum* subsp. *brasiliense* SX309 and other *Pectobacterium* spp. (XLSX 13 kb)

Additional file 18: **Figure S8.** Physical map of flagellar genes and chemotaxis genes in *P. carotovorum* subsp. *brasiliense* SX309. Arrows denote putative transcriptional units. The double slashes indicate long genetic distance. (TIF 1691 kb)

Additional file 19: **Table S11.** Identification of homologs of flagellar and chemotaxis genes in *P*. *carotovorum* subsp. *brasiliense* SX309 and other *Pectobacterium* spp. (XLSX 15 kb)

Additional file 20: **Table S12.** Homologs of lipopolysaccharide biosynthesis genes in *P*. *carotovorum* subsp. *brasiliense* SX309 and other *Pectobacterium* spp. (XLSX 14 kb)

Additional file 21: **Table S13.** Homologs of Clustered regularly interspaced short palindromic repeats (CRISPR)-CRISPR-associated protein (Cas) in *P*. *carotovorum* subsp. *brasiliense* SX309 and other *Pectobacterium* spp. (XLSX 11 kb)

Additional file 22: **Table S14.** Lists of CRISPR target viruses or plasmids based on spacer sequences among *Pectobacterium* spp. (XLSX 33 kb)

## Data Availability

GenBank BioProject: This Complete Genome project has been deposited at DDBJ/ENA/GenBank under the BioProject PRJNA379343 and link: https://www.ncbi.nlm.nih.gov/bioproject/PRJNA379343 GenBank Accession This Complete Genome project has been deposited at DDBJ/ENA/GenBank under the accessions no.: CP020350.1

## Abbreviations

AHL: N-acyl homoserine lactone
ANI: Average nucleotide identity
DDH: DNA-DNA hybridization
COG: Class of genes
LCB: Local collinear blocks
PCWDE: Plant Cell Wall Degrading Enzymes
QS: Quorum sensing
AHL: N-acyl homoserine
LPS: Lipopolysaccharide
CRISPR: Clustered regularly interspaced short palindromic repeat
Cas: CRISPR-associated sequence
