# Supplementary material for: Comparative genomic analysis of Pectobacterium carotovorum subsp. brasiliense SX309 provides novel insights into its genetic and phenotypic features
Source: BMC Genomics. 2019 Jun 13;20:486. doi: 10.1186/s12864-019-5831-x (PMC6567464; doi:10.1186/s12864-019-5831-x)
Supplement: Supplementary file 1 — Table S1. Classification and general features of Pectobacterium carotovorum subsp. brasiliense SX309 according to the MIGS recommendations. (DOCX 15 kb) [file 12864_2019_5831_MOESM1_ESM.docx]

**Table S1** Classification and general features of *Pectobacterium carotovorum* subsp. *brasiliense* SX309 according to the MIGS recommendations.

| **MIGS ID** | **Property** | **Term** | **Evidence code^a^** |
| --- | --- | --- | --- |
|  | Classification | Domain *Bacteria* | TAS |
|  |  | Phylum *Proteobacteria* | TAS |
|  |  | Class *Gammaproteobacteria* | TAS |
|  |  | Order *Enterobacteriales* | TAS |
|  |  | Family *Pectobacteriaceae* | TAS |
|  |  | Genus *Pectobacterium* | TAS |
|  |  | Species *Pectobacterium carotovorum* | TAS |
|  |  | Subspecies *brasiliense* | IDA |
|  |  | Strain SX309 | IDA |
|  | Gram stain | Negative | TAS |
|  | Cell shape | Rod-shaped | IDA |
|  | Motility | Motile | TAS |
|  | Sporulation | Non-sporulating | TAS |
|  | Temperature range | Mesophilic | TAS |
|  | Optimum temperature | 28°C | IDA |
|  | pH range; Optimum | Not reported; 7 | IDA |
|  | Carbon source | Acetic acid, α-D-glucose, D-fructose, D-mannitol, maltose, sucrose, lactose, inositol, D-cellobiose, D-sorbitol, D-melibiose, Succinic acid, Thymidine, Uridine | IDA |
| MIGS-6 | Habitat | Plants, soil | IDA |
| MIGS-6.3 | Salinity | 0.5-5% NaCl (w/v) | IDA |
| MIGS-22 | Oxygen requirement | Facultatively anaerobic | TAS |
| MIGS-15 | Biotic relationship | Free-living | TAS |
| MIGS-14 | Pathogenicity | Pathogenic | IDA |
| MIGS-4 | Geographic location | Shanxi, China | IDA |
| MIGS-5 | Sample collection | February 2015 | IDA |
| MIGS-4.1 | Latitude | 37^°^38^′^5.82^″^N | IDA |
| MIGS-4.2 | Longitude | 112^°^47^′^31.09^″^E | IDA |
| MIGS-4.4 | Altitude | Not reported | NAS |

^a^Evidence codes-IDA: Inferred from Direct Assay; TAS: Traceable Author Statement (i.e., a direct report exists in the literature); NAS: Non-traceable Author Statement (i.e., not directly observed for the living, isolated sample, but based on a generally accepted property for the species, or anecdotal evidence). These evidence codes are from the Gene Ontology project.
