# Supplementary figures and images for: Comparative genomic analysis of Pectobacterium carotovorum subsp. brasiliense SX309 provides novel insights into its genetic and phenotypic features
Source: BMC Genomics. 2019 Jun 13;20:486. doi: 10.1186/s12864-019-5831-x (PMC6567464; doi:10.1186/s12864-019-5831-x)

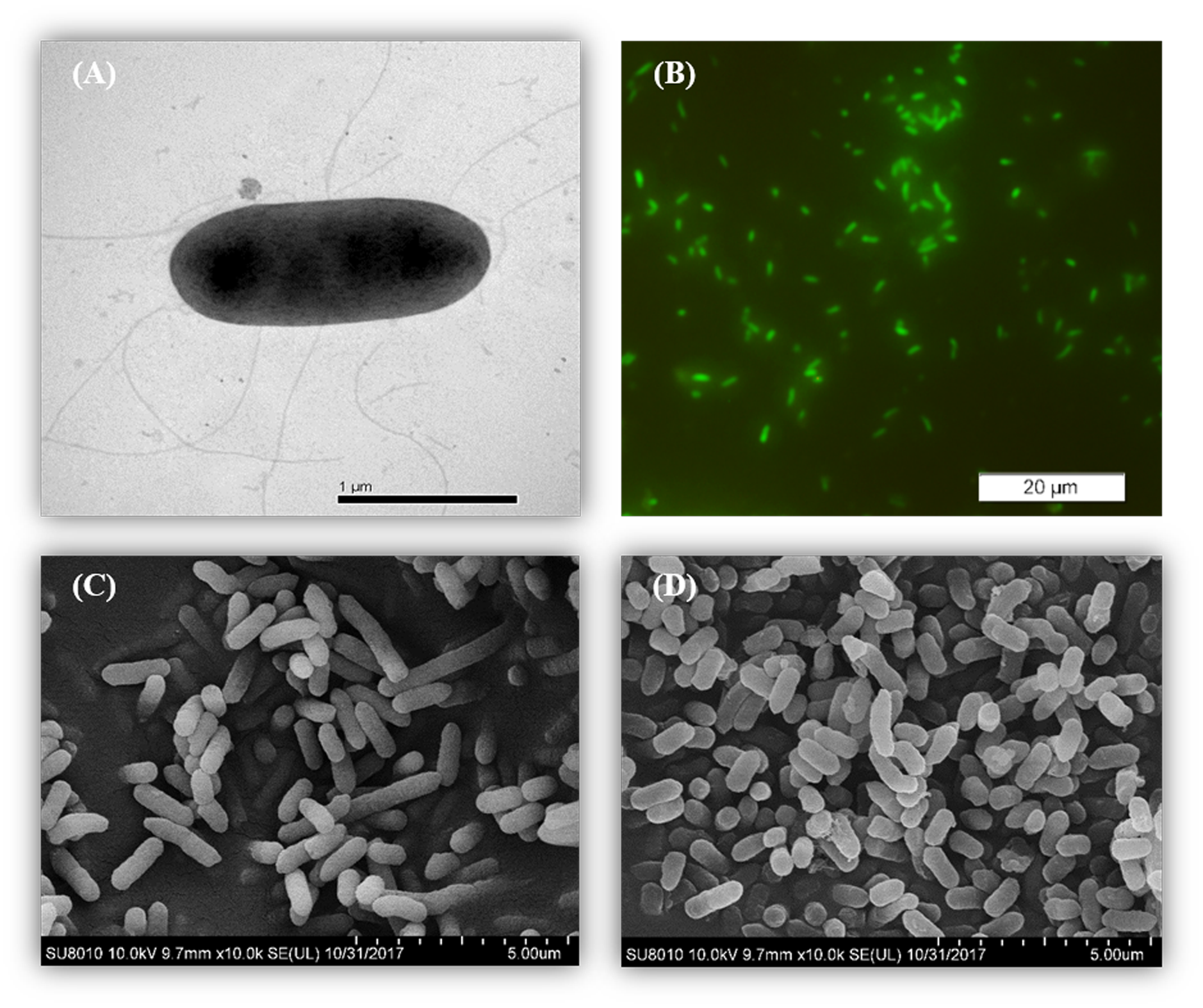

Supplement: Supplementary file 2 — Figure S1. General characteristics of P. carotovorum subsp. brasiliense SX309. Image of SX309 cells using transmission electron microscopy (A) and fluorescent microscopy (B). Image of SX309 cells from the exponential growth phase (C) and the stationary phase (D), respectively. (TIF 2925 kb) [file 12864_2019_5831_MOESM2_ESM.tif]

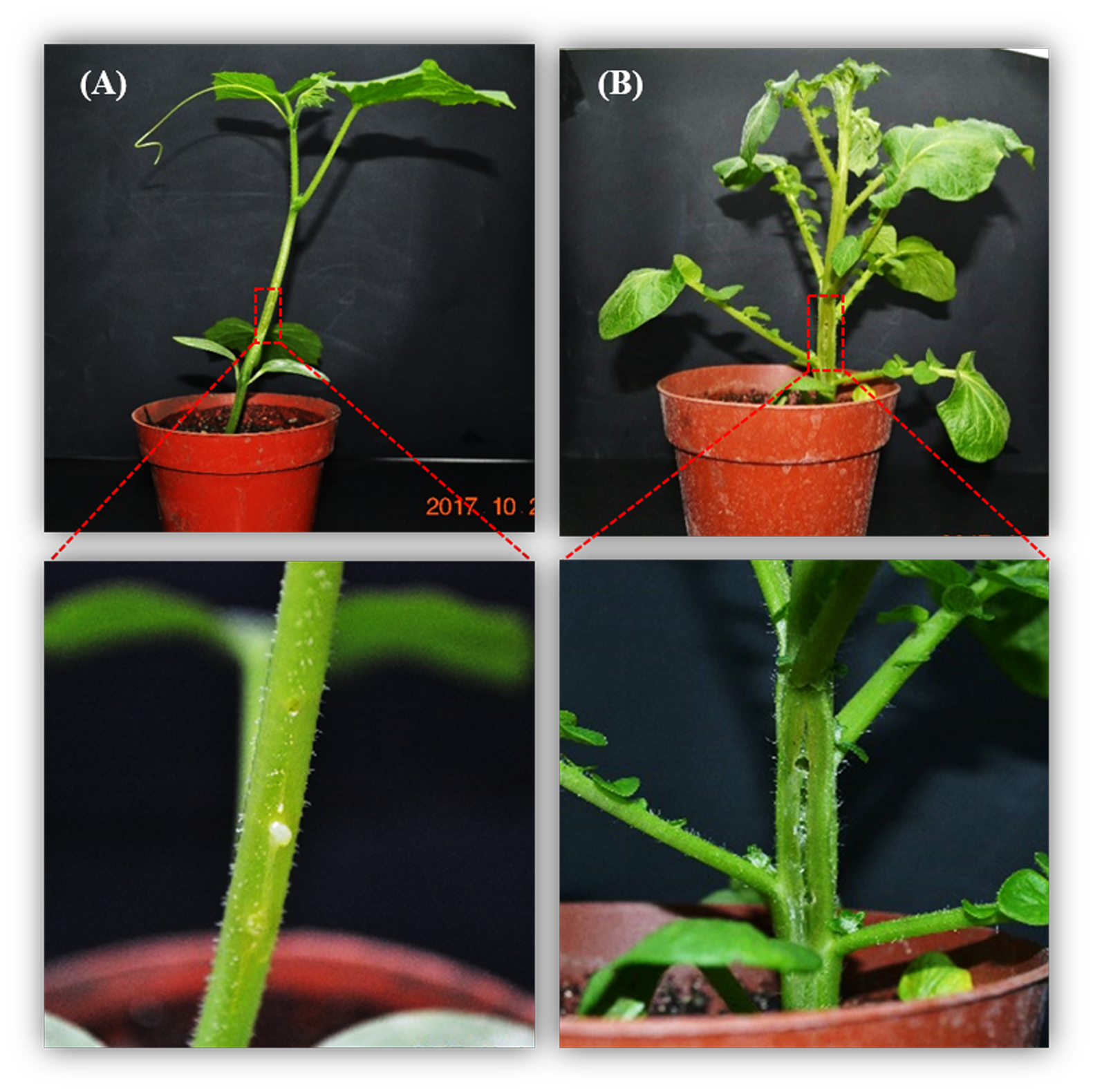

Supplement: Supplementary file 3 — Figure S2. P. carotovorum subsp. brasiliense SX309 symptoms on representative cucumber (A, Cucumis sativus) and potato (B, Solanum tuberosum) stems. The bacterial cells were used to inoculate cucumber or potato stem at 108 cfu·mL-1. At 24 hours after inoculation, the soft rot (A) or blackleg (B) symptom were observed and photographed. (TIF 3173 kb) [file 12864_2019_5831_MOESM3_ESM.tif]

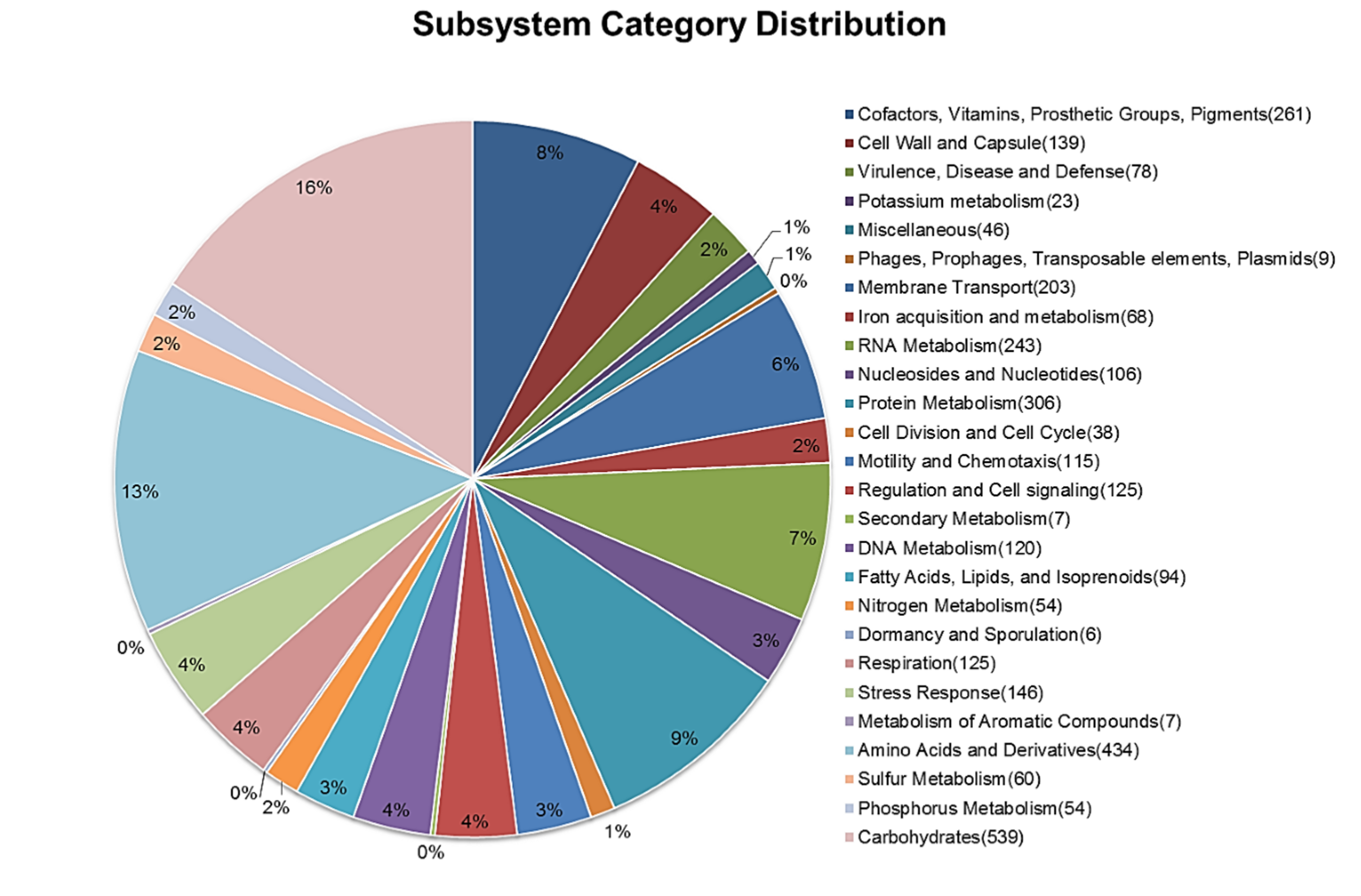

Supplement: Supplementary file 6 — Figure S3. Annotation of P. carotovorum subsp. brasiliense SX309 as generated by the Rapid Annotation using Subsystem Technology (RAST, http://rast.nmpdr.org/) webserver. (TIF 2271 kb) [file 12864_2019_5831_MOESM6_ESM.tif]

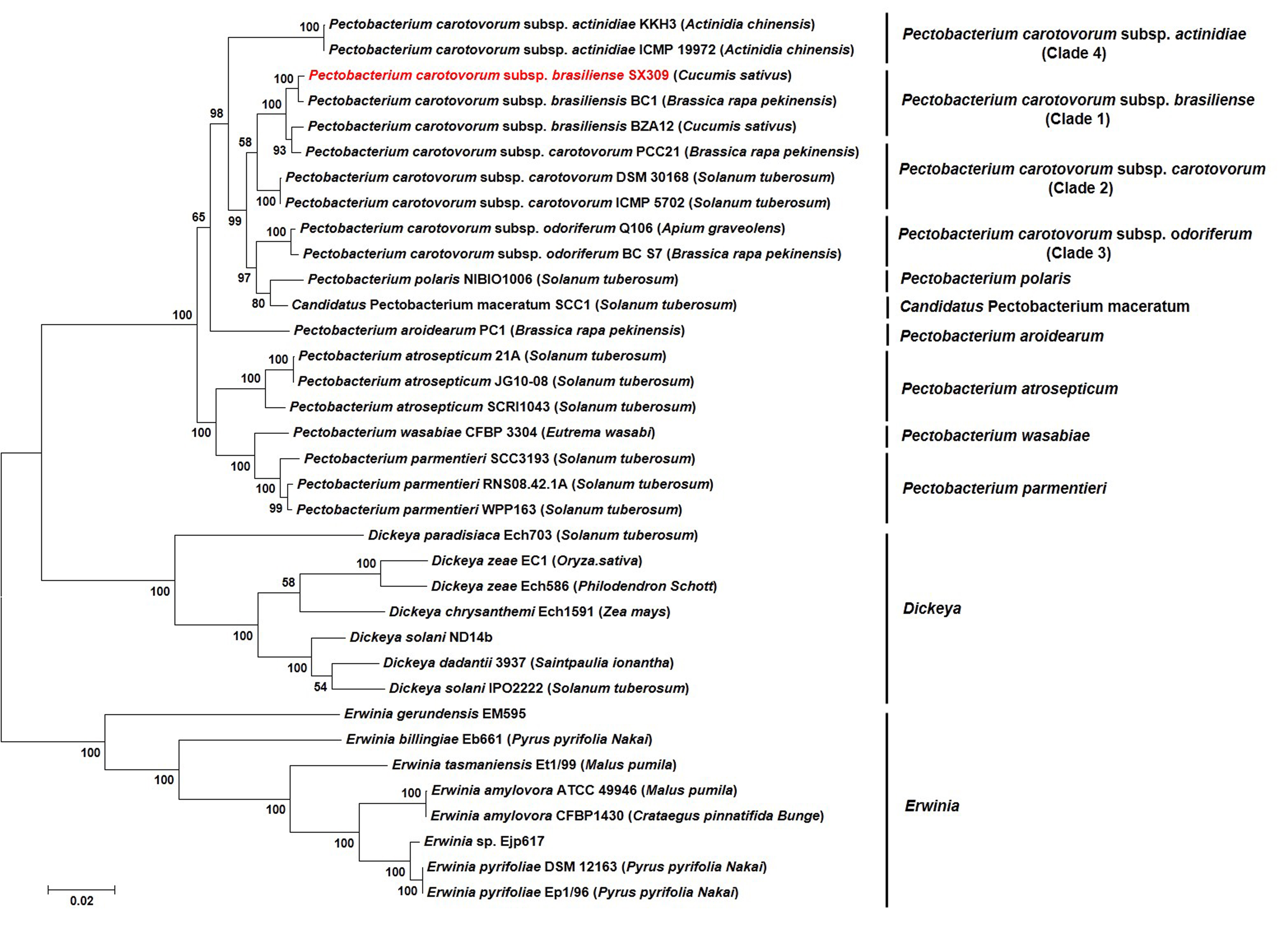

Supplement: Supplementary file 8 — Figure S4. Phylogenetic tree highlighting the relative position of P. carotovorum subsp. brasiliense SX309 within other Pectobacterium, Dickeya, and Erwinia species. The phylogenetic tree was constructed based on six housekeeping genes (16S rRNA, gapA, gyrA, atpD, rpoA, rho) according to the aligned gene sequences using maximum likelihoods derived from MEGA 6.0 software. Bootstrap values (1,000 replicates) are shown at the branch points. The scale bar indicates 0.02 nucleotide substitution per nucleotide position. The original hosts of bacteria were shown in the brackets. GenBank accession numbers associated to the housekeeping loci of all strains can be found in Additional file 3: Table S4. (TIF 4868 kb) [file 12864_2019_5831_MOESM8_ESM.tif]

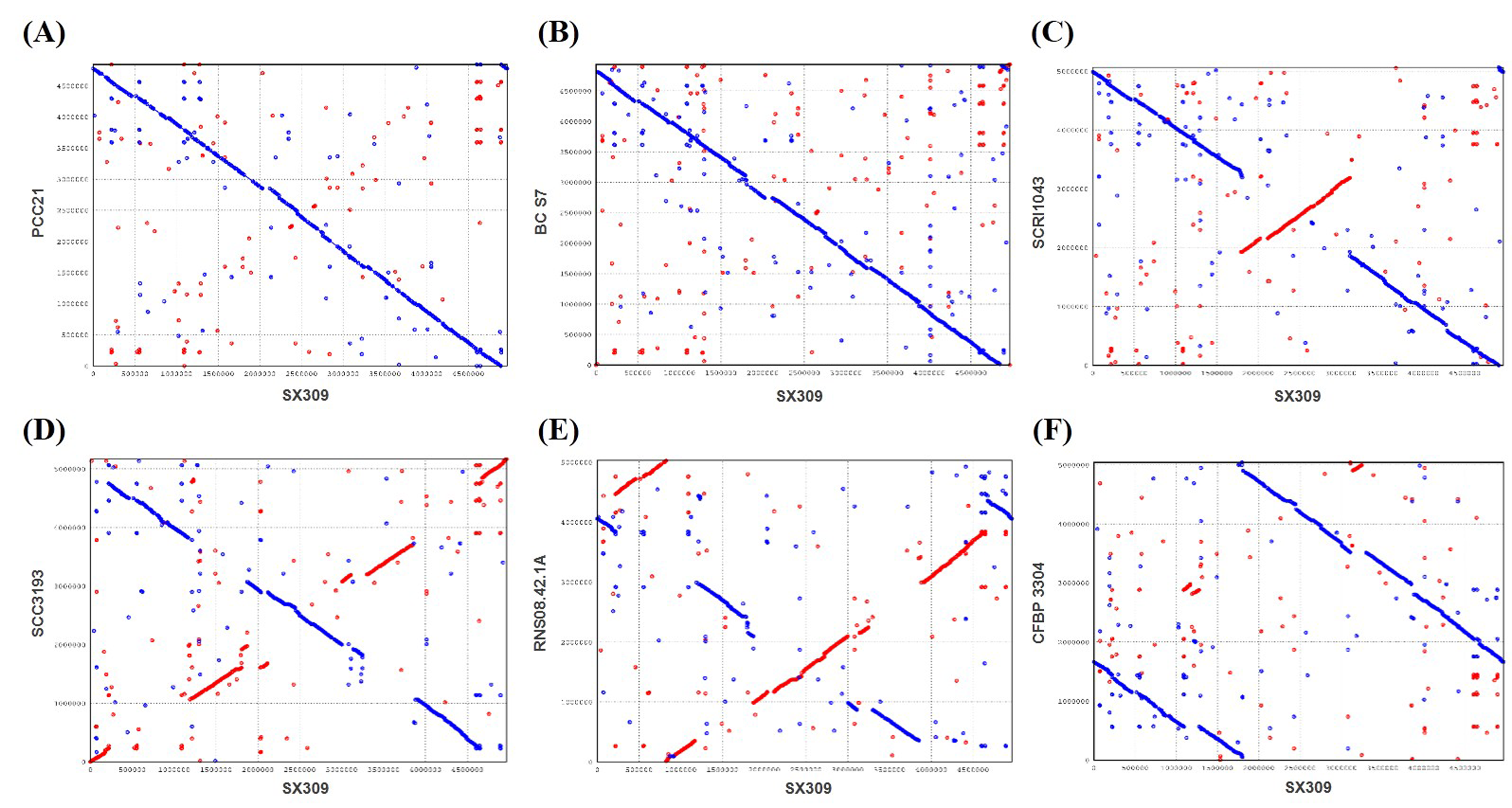

Supplement: Supplementary file 10 — Figure S5. Dot-plot analysis of linear genomic organization between P. carotovorum subsp. brasiliense SX309 and other six previously fully sequenced Pectobacterium genomes. The X-axis represents the SX309 genome, Y-axis represents PCC21 genome (A), BC S7 genome (B), SCRI1043 genome (C), SCC3193 genome (D), RNS08.42.1A genome (E), and CFBP 3304 genome (F), respectively. Red indicates the alignment sequence in the forward direction, blue indicates the alignment sequence in the reverse direction. (TIF 3562 kb) [file 12864_2019_5831_MOESM10_ESM.tif]

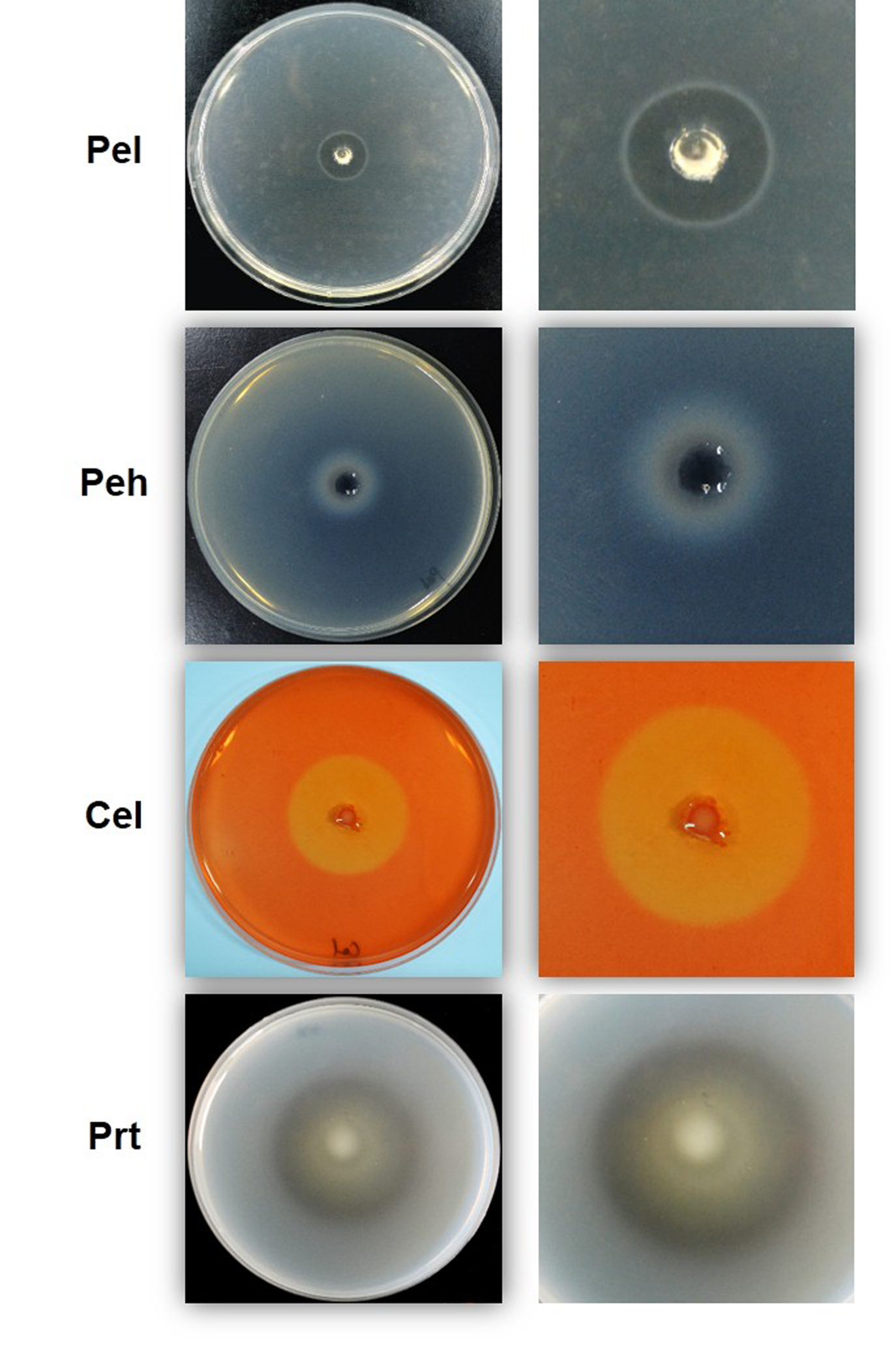

Supplement: Supplementary file 11 — Figure S6. Production of extracellular enzymes in P. carotovorum subsp. brasiliense SX309. Plate assays for the activity of pectate lyase (Pel), polygalacturonase (Peh), cellulase (Cel), and protease (Prt). In the center point of plate, wells were made in agarose media with a no. 2 cork borer and the bottoms were sealed with 0.8% (w/v) molten agarose. Bacterial cells were grown until early stationary phase at 28°C in NB medium (noninduced). After adjustment of the optical density of cell suspensions at 600 nm to 0.6 by adding sterilized distilled water, 10 μl of the cultures were applied to each well. After incubation at 28°C, each plate was treated as described in Methods. Three independent experiments had similar results. (TIF 2528 kb) [file 12864_2019_5831_MOESM11_ESM.tif]

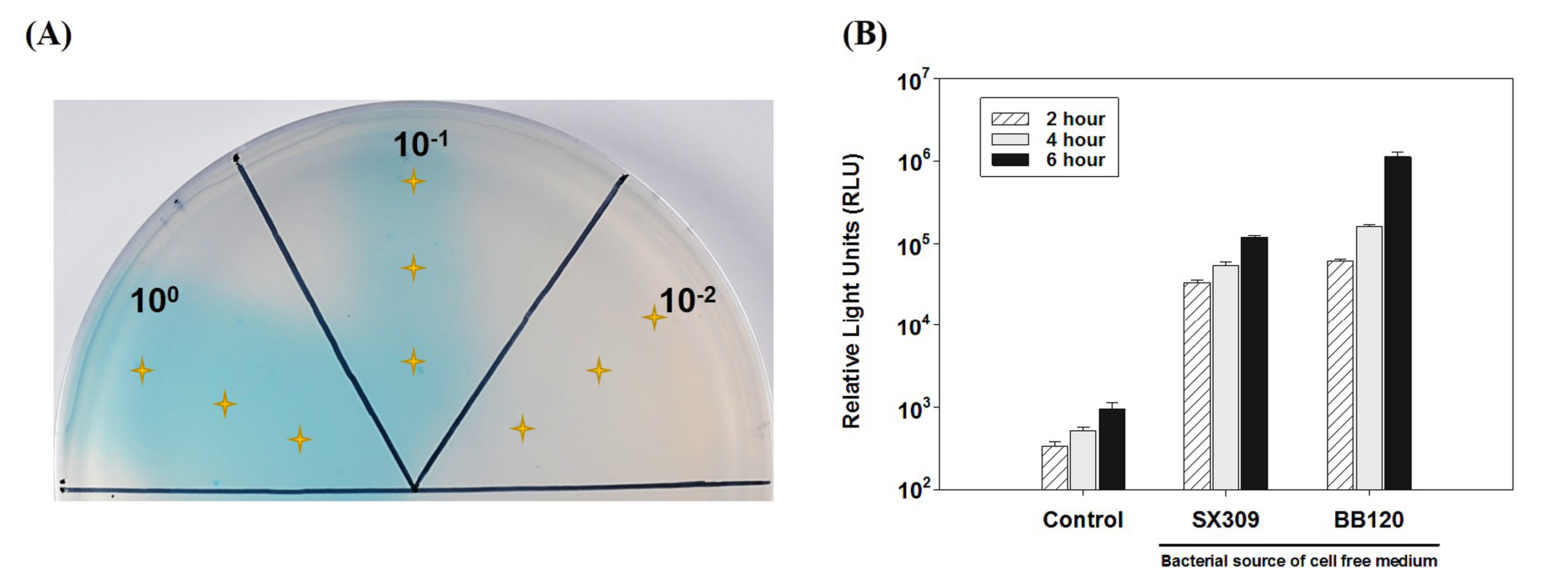

Supplement: Supplementary file 15 — Figure S7. The detection of AI-1 and AI-2 QS signal biosynthesis in P. carotovorum subsp. brasiliense SX309. (A) Analysis of N-acyl-homoserine lactone (AHL) produced by P. carotovorum subsp. brasiliense SX309. β-Galactosidase activity of the traG-lacZ fusion in the biosensor strain A. tumefaciens NTL4 (pZLR4) was measured after incubation with AHL extracted from the wild-type SX309. All experiments were performed in triplicate, and error bars indicate standard deviation; those with a different letter are significantly different according to least signification difference test (P<0.05). (B) Induction of bioluminescence in Vibrio harveyi reporter strain BB170 by cell-free medium (CFM) from P. carotovorum subsp. brasiliense SX309. Sterile AB medium and CFM from 5 mL cultures of V. harveyi BB120 were used as negative and positive controls. The baseline is the value when uninoculated (sterile) CFM alone at 2, 4, 6 h were used. Each bar represents the mean (±SD) of triplicate experiments. (TIF 1825 kb) [file 12864_2019_5831_MOESM15_ESM.tif]

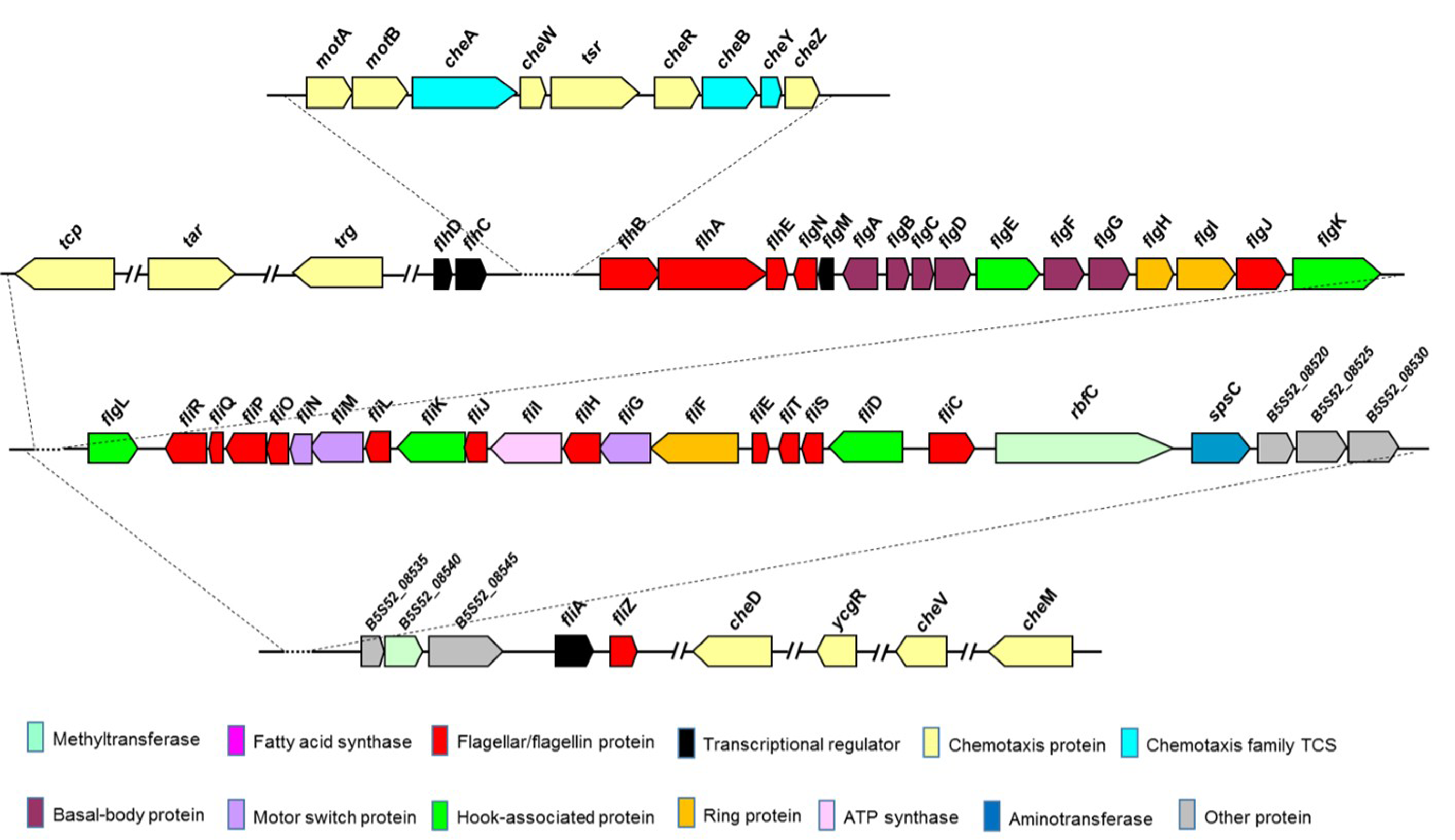

Supplement: Supplementary file 18 — Figure S8. Physical map of flagellar genes and chemotaxis genes in P. carotovorum subsp. brasiliense SX309. Arrows denote putative transcriptional units. The double slashes indicate long genetic distance. (TIF 1691 kb) [file 12864_2019_5831_MOESM18_ESM.tif]
