# Supplementary material for: Comparative genomic analysis of Pectobacterium carotovorum subsp. brasiliense SX309 provides novel insights into its genetic and phenotypic features
Source: BMC Genomics. 2019 Jun 13;20:486. doi: 10.1186/s12864-019-5831-x (PMC6567464; doi:10.1186/s12864-019-5831-x)
Supplement: Supplementary file 4 — Table S2. Project information. (DOCX 14 kb) [file 12864_2019_5831_MOESM4_ESM.docx]

**Table S2** Project information

| **MIGS ID** | **Property** | **Term** |
| --- | --- | --- |
| MIGS 31 | Finishing quality | Complete genome |
| MIGS-28 | Libraries used | PacBio 20-kb SMRT-bell library |
| MIGS 29 | Sequencing platforms | PacBio RS II |
| MIGS 31.2 | Fold coverage | 90.89-fold |
| MIGS 30 | Assemblers | PacBio SMRT Analysis software v.2.3.0 |
| MIGS 32 | Gene calling method | NCBI Prokaryotic Genome Annotation Pipeline; GeneMarkS+ |
|  | Locus Tag | B5S52 |
|  | Genbank ID | CP020350 |
|  | GenBank Date of Release | March 22, 2017 |
|  | GOLD ID | Go0375964 |
|  | BIOPROJECT | PRJNA379343 |
| MIGS 13 | Source Material Identifier | SX309 |
|  | Project relevance | Plant-bacteria interaction, vegetable pathogen |
