# Supplementary material for: Comparative genomic analysis of Pectobacterium carotovorum subsp. brasiliense SX309 provides novel insights into its genetic and phenotypic features
Source: BMC Genomics. 2019 Jun 13;20:486. doi: 10.1186/s12864-019-5831-x (PMC6567464; doi:10.1186/s12864-019-5831-x)
Supplement: Supplementary file 5 — Table S3. Genome statistics. (DOCX 12 kb) [file 12864_2019_5831_MOESM5_ESM.docx]

**Table S3** Genome statistics

| **Attribute** | **Value** | **% of total** |
| --- | --- | --- |
| Genome size (bp) | 4,966,299 | 100.00 |
| DNA coding (bp) | 4,275,399 | 86.09 |
| DNA G + C (bp) | 2,591,415 | 52.18 |
| DNA scaffolds | 1 | 100.00 |
| Total genes | 4455 | 100.00 |
| Protein coding genes | 4252 | 95.44 |
| RNA genes | 104 | 2.33 |
| Pseudo genes | 99 | 2.22 |
| Genes in internal clusters | ^a^ND | - |
| Genes with function prediction | 3118 | 69.99 |
| Genes assigned to COGs | 3474 | 77.98 |
| Genes with Pfam domains | 3849 | 86.40 |
| Genes with signal peptides | 409 | 9.18 |
| Genes with transmembrane helices | 19 | 0.43 |
| CRISPR repeats | 3 | - |

^a^ND = not determined
