# Supplementary material for: Comparative genomic analysis of Pectobacterium carotovorum subsp. brasiliense SX309 provides novel insights into its genetic and phenotypic features
Source: BMC Genomics. 2019 Jun 13;20:486. doi: 10.1186/s12864-019-5831-x (PMC6567464; doi:10.1186/s12864-019-5831-x)
Supplement: Supplementary file 7 — Table S4. The locus tag information of 16S rRNA genes and five housekeeping genes used for phylogenetic analysis in this study. (DOCX 19 kb) [file 12864_2019_5831_MOESM7_ESM.docx]

**Table S4** The locus tag information of 16S rRNA genes and five housekeeping genes used for phylogenetic analysis in this study.

| **Strain**  **(^a^Name)** | **16S rRNA**  **16S ribosomal RNA** | ***gap*A**  **Glyceraldehyde-3-phosphate dehydrogenase** | ***gyr*A**  **DNA gyrase subunit A** | ***atp*D**  **ATP synthase chain beta** | ***rpo*A**  **DNA-directed RNA polymerase, subunit alpha** | ***rho***  **Transcription termination factor Rho** |
| --- | --- | --- | --- | --- | --- | --- |
| SX309 | B5S52_02585 | B5S52_11815 | B5S52_15995 | B5S52_22070 | B5S52_02510 | B5S52_01150 |
| BC1 | NC16_16860 | NC16_10010 | NC16_05460 | NC16_21345 | NC16_19015 | NC16_20030 |
| BZA12 | CTV95_13730 | CTV95_04540 | CTV95_08950 | CTV95_15110 | CTV95_17635 | CTV95_16480 |
| PC1 | PC1_R0067 | PC1_1965 | PC1_1965 | PC1_4257 | PC1_3797 | PC1_4011 |
| PCC21 | PCC21_rRNA000200 | PCC21_020260 | PCC21_042390 | PCC21_042390 | PCC21_037850 | PCC21_039900 |
| BC S7 | BCS7_00865 | BCS7_10145 | BCS7_05730 | BCS7_21390 | BCS7_18930 | BCS7_20060 |
| Q106 | KT343626 | BV921_13355 | BV921_09485 | BV921_16725 | BV921_22205 | BV921_18195 |
| ICMP 5702 | G032_18350 | G032_12315 | G032_05770 | G032_20745 | G032_18375 | G032_19445 |
| DSM 30168 | NR_041971 | SAMN05444147_101280 | SAMN05444147_103367 | SAMN05444147_107231 | SAMN05444147_11815 | SAMN05444147_108192 |
| KKH3 | NR_125539 | KKH3_19450 | KKH3_09580 | KKH3_42220 | KKH3_37100 | KKH3_39650 |
| ICMP 19972 | BSK71_08965 | BSK71_13255 | BSK71_03400 | BSK71_18250 | BSK71_20780 | BSK71_16715 |
| SCC1 | SCC1_0194 | SCC1_2178 | SCC1_3166 | SCC1_4402 | SCC1_0439 | SCC1_0231 |
| SCC3193 | W5S_3637 | W5S_2202 | W5S_3209 | W5S_4734 | W5S_4136 | W5S_4366 |
| CFBP 3304 | A7983_06320 | A7983_18675 | A7983_18675 | A7983_07720 | A7983_05005 | A7983_06160 |
| SCRI1043 | 16S_rRNA-1 | ECA2344 | ECA1201 | ECA4512 | ECA4006 | ECA4211 |
| JG10-08 | EV46_19855 | EV46_11280 | EV46_0604 | EV46_22460 | EV46_19930 | EV46_21005 |
| 21A | GZ59_43070 | GZ59_22140 | GZ59_12300 | GZ59_46110 | GZ59_40620 | GZ59_42750 |
| WPP163 | Pecwa_R0095 | Pecwa_2261 | Pecwa_3211 | Pecwa_454 | Pecwa_3975 | Pecwa_3975 |
| RNS08.42.1A | A8F97_17225 | A8F97_07325 | A8F97_02595 | A8F97_18700 | A8F97_21605 | A8F97_20465 |
| NIBIO1006 | BJJ97_00415 | BJJ97_15115 | BJJ97_10920 | BJJ97_04970 | BJJ97_02650 | BJJ97_03670 |
| EC1 | W909_01010 | W909_09290 | W909_05450 | W909_20185 | W909_1805 | W909_19005 |
| Ech586 | Dd586_R0001 | Dd586_2162 | Dd586_1081 | Dd586_4159 | Dd586_3711 | Dd586_3900 |
| IPO2222 | A4U42_03735 | A4U42_19205 | A4U42_14785 | A4U42_08840 | A4U42_06715 | A4U42_07640 |
| ND14b | LH89_10945 | LH89_01940 | LH89_06440 | LH89_12580 | LH89_14780 | LH89_13845 |
| Ech1591 | Dd1591_R0100 | Dd1591_2215 | Dd1591_3019 | Dd1591_4194 | Dd1591_0348 | Dd1591_0157 |
| 3937 | Dda3937_04220 | Dda3937_03335 | Dda3937_01774 | Dda3937_00145 | Dda3937_01515 | Dda3937_00267 |
| Ech703 | Dd703_R0005 | Dd703_1973 | Dd703_1039 | Dd703_3993 | Dd703_0430 | Dd703_0204 |
| CFBP1430 | EAMY_r01 | EAMY_1976 | EAMY_2345 | EAMY_3702 | EAMY_3361 | EAMY_0170 |
| ATCC 49946 | EAM_r001 | EAM_1931 | EAM_2264 | EAM_3474 | EAM_3173 | EAM_0163 |
| Ep1/96 | EpC_r22 | EpC_16320 | EpC_12850 | EpC_36910 | EpC_33500 | EpC_01840 |
| Eb661 | EbC_08400_tr07 | EbC_24210 | EbC_30400 | EbC_45870 | EbC_41050 | EbC_01950 |
| Et1/99 | ETA_r010 | ETA_15590 | ETA_12260 | ETA_34750 | ETA_31380 | ETA_01960 |
| Ejp617 | EJP617_r003 | EJP617_30660 | EJP617_34080 | EJP617_11650 | EJP617_08420 | EJP617_13820 |
| DSM 12163 | EPYR_r001 | EPYR_01755 | EPYR_01369 | EPYR_03975 | EPYR_03610 | EPYR_00193 |
| EM595 | EM595_r001 | EM595_2009 | EM595_2486 | EM595_3461 | EM595_0393 | EM595_3337 |

**^a^Name:** SX309: *Pectobacterium carotovorum* subsp. *brasiliense* SX309; BC1: *Pectobacterium carotovorum* subsp. *brasiliensis* BC1; BZA12: *Pectobacterium carotovorum* subsp. *brasiliensis* BZA12; PC1: *Pectobacterium aroidearum* PC1; PCC21: *Pectobacterium carotovorum* subsp. *carotovorum* PCC21; BC S7: *Pectobacterium carotovorum* subsp. *odoriferum* BC S7; Q106: *Pectobacterium carotovorum* subsp. *odoriferum* Q106; ICMP 5702: *Pectobacterium carotovorum* subsp. *carotovorum* ICMP 5702; DSM 30168: *Pectobacterium carotovorum* subsp. *carotovorum* DSM 30168; KKH3: *Pectobacterium carotovorum* subsp. *actinidiae* KKH3; ICMP 19972: *Pectobacterium carotovorum* subsp. *actinidiae* ICMP 19972; SCC1: *Candidatus* Pectobacterium maceratum SCC1; SCC3193: *Pectobacterium* *parmentieri* SCC3193; CFBP 3304: *Pectobacterium wasabiae* CFBP 3304; SCRI1043: *Pectobacterium atrosepticum* SCRI1043; JG10-08: *Pectobacterium atrosepticum* JG10-08; 21A: *Pectobacterium atrosepticum* 21A; WPP163: *Pectobacterium parmentieri* WPP163; RNS08.42.1A: *Pectobacterium parmentieri* RNS08.42.1A; NIBIO1006: *Pectobacterium polaris* NIBIO1006; EC1: *Dickeya zeae* EC1; Ech586: *Dickeya zeae* Ech586; IPO2222: *Dickeya solani* IPO2222; ND14b: *Dickeya solani* ND14b; Ech1591: *Dickeya chrysanthemi* Ech1591; 3937: *Dickeya dadantii* 3937; Ech703: *Dickeya paradisiaca* Ech703; CFBP1430: *Erwinia amylovora* CFBP1430; ATCC 49946: *Erwinia amylovora* ATCC 49946; Ep1/96: *Erwinia pyrifoliae* Ep1/96; Eb661: *Erwinia billingiae* Eb661; Et1/99: *Erwinia tasmaniensis* Et1/99; Ejp617: *Erwinia* sp. Ejp617; DSM 12163: *Erwinia pyrifoliae* DSM 12163; EM595: *Erwinia gerundensis* EM595.
