# Supplementary material for: Comparative genomic analysis of Pectobacterium carotovorum subsp. brasiliense SX309 provides novel insights into its genetic and phenotypic features
Source: BMC Genomics. 2019 Jun 13;20:486. doi: 10.1186/s12864-019-5831-x (PMC6567464; doi:10.1186/s12864-019-5831-x)
Supplement: Supplementary file 12 — Table S6. Homologs of cell wall-degrading enzyme genes in P. carotovorum subsp. brasiliense SX309 and other Pectobacterium spp. (DOCX 23 kb) [file 12864_2019_5831_MOESM12_ESM.docx]

**Table S6** Homologs of cell wall-degrading enzyme genes in *P. carotovorum* subsp. *brasiliense* SX309 and other *Pectobacterium* spp.

| **Genes in SX309^*^** | **Accesion**  **no. in SX309** | **PCC21** |  | **SCC3193** |  | **SCRI1043** |  | **RNS08.42.1A** |  |
| --- | --- | --- | --- | --- | --- | --- | --- | --- | --- |
|  |  | **Accesion**  **no.** | **Homology**  **(%)** | **Accesion**  **no.** | **Homology**  **(%)** | **Accesion**  **no.** | **Homology**  **(%)** | **Accesion**  **no.** | **Homology**  **(%)** |
| **Pectate lyase** |  |  |  |  |  |  |  |  |  |
| *pel*N (B5S52_12825) | ^a^ND | AFR03183.1 | 99 | AFI90081.1 | 98 | CAG75452.1 | 97 | AOR58972.1 | 98 |
| *pel*I (B5S52_16570) | ARA77413.1 | AFR02426.1 | 100 | AFI91393.1 | 95 | CAG74004.1 | 94 | AOR57694.1 | 95 |
| *pel*A (B5S52_01920) | ARA74711.1 | AFR05253.1 | 100 | AFI92248.1 | 96 | CAG76964.1 | 98 | AOR61396.1 | 96 |
| *pel*Y (B5S52_10850) | ARA76346.1 | AFR03652.1 | 99 | AFI90572.1 | 97 | CAG75037.1 | 97 | AOR58476.1 | 97 |
| *pel*C (B5S52_01910) | ARA74709.1 | AFR05255.1 | 100 | AFI92250.1 | 98 | CAG76966.1 | 96 | AOR61394.1 | 98 |
| *pel*B (B5S52_01915) | ARA74710.1 | AFR05254.1 | 100 | AFI92249.1 | 94 | CAG76965.1 | 92 | AOR61395.1 | 94 |
| *pel*Z (B5S52_01905) | ARA74708.1 | AFR05256.1 | 100 | AFI92251.1 | 95 | CAG76967.1 | 97 | AOR61393.1 | 95 |
| *pel*W (B5S52_12135) | ARA76585.1 | AFR03371.1 | 99 | AFI90232.1 | 94 | CAG75305.1 | 98 | AOR58772.1 | 95 |
| *pel*X (B5S52_22085) | ARA78409.1 | AFR05640.1 | 99 | AFI92788.1 | 97 | CAG77405.1 | 96 | AOR60892.1 | 97 |
| **Pectin lyase** |  |  |  |  |  |  |  |  |  |
| *pnl* (B5S52_14620) | ARA77053.1 | AFR02806.1 | 100 | AFI89782.1 | 95 | CAG74408.1 | 94 | AOR59222.1 | 95 |
| **Pectinesterase** |  |  |  |  |  |  |  |  |  |
| *pem*A (B5S52_06380) | ARA75515.1 | AFR04488.1 | 99 | AFI89239.1 | 88 | CAG76151.1 | 88 | AOR59761.1 | 88 |
| *pem*B (B5S52_21415) | ARA78286.1 | AFR01499.1 | 97 | AFI92651.1 | 88 | CAG73027.1 | 87 | AOR61021.1 | 87 |
| **Pectin acetylesterase** |  |  |  |  |  |  |  |  |  |
| *Pae*X (B5S52_12165) | ARA76591.1 | AFR03365.1 | 98 | AFI90225.1 | 93 | CAG75311.1 | 94 | AOR58778.1 | 93 |
| *pae*Y (B5S52_06385) | ARA75516.1 | AFR04487.1 | 99 | AFI89240.1 | 93 | CAG76150.1 | 92 | AOR59760.1 | 93 |
| **Polygalacturonases** |  |  |  |  |  |  |  |  |  |
| *peh*X (B5S52_07160) | ARA75661.1 | AFR04338.1 | 99 | AFI89393.1 | 95 | CAG76010.1 | 97 | AOR59588.1 | 95 |
| *peh*N (B5S52_16060) | ARA77313.1 | AFR02527.1 | 99 | AFI91295.1 | 99 | CAG74100.1 | 99 | AOR57790.1 | 99 |
| *peh*A (B5S52_16565) | ARA77412.1 | AFR02427.1 | 100 | AFI91392.1 | 96 | CAG74005.1 | 96 | AOR57695.1 | 96 |
| *peh*K (B5S52_04800) | ARA75245.1 | AFR04780.1 | 99 | ^b^NA | ^b^NA | CAG76450.1 | 96 | ^b^NA | ^b^NA |
| **Oligogalacturonide lyase** |  |  |  |  |  |  |  |  |  |
| *ogl* (B5S52_12255) | ARA76609.1 | AFR03347.1 | 99 | AFI90206.1 | 98 | CAG75329.1 | 98 | AOR58797.1 | 98 |
| **Rhamnogalacturonate lyase** |  |  |  |  |  |  |  |  |  |
| *rhi*E (B5S52_18125) | ARA77692.1 | AFR02118.1 | 98 | AFI88919.1 | 92 | CAG73718.1 | 92 | AOR60073.1 | 92 |
| **Cellulases** |  |  |  |  |  |  |  |  |  |
| *bcs*C (B5S52_00440) | ARA78447.1 | AFR05537.1 | 97 | AFI88210.1 | 91 | CAG77270.1 | 90 | AOR60769.1 | 91 |
| *bcs*Z (B5S52_00445) | ARA74438.1 | AFR05536.1 | 97 | AFI88211.1 | 87 | CAG77269.1 | 92 | AOR60768.1 | 87 |
| *bcs*B (B5S52_00450) | ARA74439.1 | AFR05535.1 | 96 | AFI88212.1 | 90 | CAG77268.1 | 92 | AOR60767.1 | 90 |
| *bcs*A (B5S52_00455) | ARA74440.1 | AFR05534.1 | 99 | AFI88213.1 | 95 | ^b^NA | ^b^NA | AOR60766.1 | 95 |
| *bcs*Q (B5S52_00460) | ARA74441.1 | AFR05533.1 | 99 | AFI88214.1 | 96 | CAG77266.1 | 96 | AOR60765.1 | 95 |
| *bcs*R (B5S52_00465) | ARA74442.1 | AFR05532.1 | 96 | AFI88215.1 | 93 | CAG77265.1 | 90 | AOR60764.1 | 93 |
| *bcs*E (B5S52_00470) | ARA74443.1 | AFR05531.1 | 97 | AFI88216.1 | 87 | CAG77263.1 | 90 | AOR60763.1 | 87 |
| *bcs*F (B5S52_00475) | ARA74444.1 | AFR05530.1 | 99 | AFI88217.1 | 93 | CAG77262.1 | 94 | AOR60762.1 | 93 |
| *bcs*G (B5S52_00480) | ARA74445.1 | AFR05529.1 | 96 | AFI88218.1 | 91 | CAG77261.1 | 92 | AOR60761.1 | 91 |
| *cel*V (B5S52_10115) | ARA76210.1 | AFR03842.1 | 97 | AFI90670.1 | 95 | CAG74882.1 | 96 | AOR58379.1 | 95 |
| *cel*H (B5S52_04340) | ARA75159.1 | AFR04873.1 | 99 | ^b^NA | ^b^NA | CAG76544.1 | 98 | ^b^NA | ^b^NA |
| *bgl*A (B5S52_00060) | ARA74369.1 | AFR05597.1 | 99 | AFI88150.1 | 96 | CAG77328.1 | 95 | AOR60827.1 | 98 |
| *bgl*B (B5S52_18905) | ARA77832.1 | AFR01964.1 | 99 | AFI88796.1 | 96 | CAG73576.1 | 97 | AOR60213.1 | 97 |
| *bgl*D (B5S52_14770) | ARA77078.1 | AFR02786.1 | 99 | AFI90743.1 | 55 | CAG77283.1 | 56 | AOR58309.1 | 55 |
| *nag*Z (B5S52_08920) | ARA75986.1 | AFR04000.1 | 99 | AFI90859.1 | 96 | CAG74717.1 | 96 | AOR58198.1 | 96 |
| *lfa*A (B5S52_10050) | ARA76197.1 | AFR03854.1 | 99 | AFI90685.1 | 94 | CAG74870.1 | 94 | AOR58366.1 | 94 |
| **Proteases** |  |  |  |  |  |  |  |  |  |
| *prt1* (B5S52_06595) | ARA75556.1 | AFR04445.1 | 99 | AFI89277.1 | 94 | CAG76109.1 | 94 | AOR59723.1 | 94 |
| *prt*C (B5S52_18630) | ARA77787.1 | AFR02025.1 | 99 | AFI88843.1 | 99 | CAG73636.1 | 99 | AOR60167.1 | 99 |
| *prt*W (B5S52_13760) | ARA76894.1 | AFR02980.1 | 100 | AFI90977.1 | 91 | CAG75685.1 | 87 | AOR58089.1 | 91 |
| *clp*S (B5S52_13145) | ARA76776.1 | AFR03118.1 | 100 | AFI90012.1 | 100 | CAG75557.1 | 96 | AOR59038.1 | 100 |
| *clp*A (B5S52_13140) | ARA76775.1 | AFR03119.1 | 100 | AFI90013.1 | 99 | CAG75556.1 | 99 | AOR59037.1 | 99 |
| *clp*X (B5S52_16275) | ARA77354.1 | AFR02484.1 | 100 | AFI91334.1 | 98 | CAG74059.1 | 98 | AOR57751.1 | 98 |
| *clp*P (B5S52_16280) | ARA77355.1 | AFR02483.1 | 100 | AFI91335.1 | 99 | CAG74058.1 | 99 | AOR57750.1 | 99 |
| *lon* (B5S52_08605) | ARA75926.1 | AFR04059.1 | 99 | AFI90927.1 | 95 | CAG74654.1 | 95 | AOR58135.1 | 95 |
| *htp*X (B5S52_12260) | ARA76610.1 | AFR03346.1 | 100 | AFI90205.1 | 97 | CAG75330.1 | 99 | AOR58798.1 | 97 |
| *soh*B (B5S52_11575) | ARA78531.1 | AFR03474.1 | 99 | AFI90347.1 | 99 | CAG75191.1 | 97 | AOR58665.1 | 99 |
| *rse*P (B5S52_16780) | ARA77453.1 | AFR02384.1 | 100 | AFI91426.1 | 97 | CAG73949.1 | 98 | AOR57663.1 | 97 |
| *hfl*B (B5S52_18750) | ARA77802.1 | AFR01995.1 | 100 | AFI88826.1 | 92 | CAG73612.1 | 98 | AOR60183.1 | 91 |
| *hfl*C (B5S52_02940) | ARA74898.1 | AFR05108.1 | 99 | AFI92125.1 | 97 | CAG76828.1 | 97 | AOR61515.1 | 97 |
| *hfl*K (B5S52_02935) | ARA74897.1 | AFR05109.1 | 100 | AFI92126.1 | 96 | CAG76829.1 | 98 | AOR61514.1 | 96 |
| *deg*P (B5S52_06110) | ARA75467.1 | AFR04535.1 | 99 | AFI89192.1 | 95 | CAG76199.1 | 96 | AOR59809.1 | 95 |
| *deg*Q (B5S52_20330) | ARA78095.1 | AFR01700.1 | 100 | AFI88437.1 | 98 | CAG73223.1 | 99 | AOR60555.1 | 98 |
| *pmb*A (B5S52_20435) | ARA78114.1 | AFR01679.1 | 99 | AFI88416.1 | 98 | CAG73202.1 | 98 | AOR60576.1 | 98 |
| *tld*D (B5S52_20470) | ARA78121.1 | AFR01671.1 | 99 | AFI88410.1 | 96 | CAG73193.1 | 97 | AOR60582.1 | 96 |
| *hsl*U (B5S52_00855) | ARA74516.1 | AFR05449.1 | 100 | AFI88321.1 | 99 | CAG77159.1 | 98 | AOR60671.1 | 99 |
| *hsl*V (B5S52_00860) | ARA74517.1 | ^b^NA | ^b^NA | AFI88322.1 | 100 | CAG77158.1 | 99 | AOR60670.1 | 100 |
| *com*M (B5S52_01050) | ARA74546.1 | AFR05416.1 | 99 | AFI92438.1 | 98 | CAG77127.1 | 97 | AOR61214.1 | 98 |
| *glp*G (B5S52_01525) | ARA74634.1 | AFR05325.1 | 99 | AFI92338.1 | 93 | CAG77035.1 | 95 | AOR61310.1 | 92 |
| *loi*P (B5S52_03015) | ARA78466.1 | AFR05093.1 | 99 | AFI92110.1 | 94 | CAG76813.1 | 95 | AOR61529.1 | 94 |

^a^ND = not determined; ^b^NA = not available.
