# Supplementary material for: Comparative genomic analysis of Pectobacterium carotovorum subsp. brasiliense SX309 provides novel insights into its genetic and phenotypic features
Source: BMC Genomics. 2019 Jun 13;20:486. doi: 10.1186/s12864-019-5831-x (PMC6567464; doi:10.1186/s12864-019-5831-x)
Supplement: Supplementary file 13 — Table S7. Identification of homologs of type II, III and Sec-SRP secretion system genes in P. carotovorum subsp. brasiliense SX309 and other Pectobacterium spp. (DOCX 25 kb) [file 12864_2019_5831_MOESM13_ESM.docx]

**Table S7** Identification of homologs of type II, III and Sec-SRP secretion system genes in *P. carotovorum* subsp. *brasiliense* SX309 and other *Pectobacterium* spp.

| **Genes in SX309^*^** | **Accesion** | **PCC21** | | **BC S7** | | **SCC3193** | | **SCRI1043** | | **RNS08.42.1A** | |
| --- | --- | --- | --- | --- | --- | --- | --- | --- | --- | --- | --- |
|  | **no. in SX309** | **Accesion** | **Homology** | **Accesion** | **Homology** | **Accesion** | **Homology** | **Accesion** | **Homology** | **Accesion** | **Homology** |
|  |  | **no.** | **(%)** | **no.** | **(%)** | **no.** | **(%)** | **no.** | **(%)** | **no.** | **(%)** |
| **Type II secretion system** | | |  |  |  |  |  |  |  |  |  |
| *hof*C (B5S52_03610) | ARA75021.1 | AFR04974.1 | 98 | AIU89729.1 | 95 | AFI91967.1 | 91 | CAG76699.1 | 91 | AOR61663.1 | 91 |
| *hof*B (B5S52_03615) | ARA75022.1 | AFR04973.1 | 98 | AIU89728.1 | 93 | AFI91966.1 | 91 | CAG76698.1 | 92 | AOR61664.1 | 91 |
| *gsp*C (B5S52_07165) | ARA75662.1 | AFR04337.1 | 99 | ^a^NA | ^a^NA | AFI89394.1 | 92 | CAG76009.1 | 95 | AOR59587.1 | 92 |
| *gsp*D (B5S52_07170) | ARA75663.1 | AFR04336.1 | 99 | AIU89204.1 | 96 | AFI89395.1 | 95 | CAG76008.1 | 96 | AOR59586.1 | 96 |
| *gsp*E (B5S52_07175) | ARA75664.1 | AFR04335.1 | 99 | AIU89203.1 | 98 | AFI89396.1 | 96 | CAG76007.1 | 96 | AOR59585.1 | 96 |
| *gsp*F (B5S52_07180) | ARA75665.1 | AFR04334.1 | 99 | AIU89202.1 | 98 | AFI89397.1 | 97 | CAG76006.1 | 97 | AOR59584.1 | 97 |
| *gsp*G (B5S52_07185) | ARA75666.1 | AFR04333.1 | 100 | AIU90534.1 | 98 | AFI89398.1 | 97 | CAG76005.1 | 90 | AOR59583.1 | 97 |
| *gsp*H (B5S52_07190) | ARA75667.1 | AFR04332.1 | 97 | AIU89201.1 | 96 | AFI89399.1 | 90 | CAG76004.1 | 91 | AOR59582.1 | 90 |
| *gsp*I (B5S52_07195) | ARA75668.1 | AFR04331.1 | 99 | AIU89200.1 | 98 | AFI89400.1 | 97 | CAG76003.1 | 94 | AOR59581.1 | 97 |
| *gsp*J (B5S52_07200) | ARA75669.1 | AFR04330.1 | 98 | AIU90533.1 | 97 | AFI89401.1 | 89 | CAG76002.1 | 92 | AOR59580.1 | 94 |
| *gsp*K (B5S52_07205) | ARA75670.1 | AFR04329.1 | 99 | AIU89199.1 | 97 | AFI89402.1 | 93 | CAG76001.1 | 95 | AOR59579.1 | 93 |
| *gsp*L (B5S52_07210) | ARA75671.1 | AFR04328.1 | 99 | AIU89198.1 | 95 | AFI89403.1 | 92 | CAG76000.1 | 95 | AOR59578.1 | 92 |
| *gsp*M (B5S52_07215) | ARA75672.1 | AFR04327.1 | 99 | AIU89197.1 | 93 | AFI89404.1 | 88 | CAG75999.1 | 87 | AOR59577.1 | 88 |
| *gsp*N (B5S52_07220) | ARA75673.1 | AFR04326.1 | 97 | AIU89196.1 | 94 | ^a^NA | ^a^NA | CAG75998.1 | 91 | ^a^NA | ^a^NA |
| *out*O (B5S52_07225) | ARA75674.1 | AFR04325.1 | 97 | ^a^NA | ^a^NA | AFI89405.1 | 88 | CAG75997.1 | 92 | AOR59576.1 | 88 |
| *out*S (B5S52_07145) | ARA75658.1 | AFR04341.1 | 100 | AIU89207.1 | 96 | AFI89391.1 | 88 | CAG76013.1 | 92 | AOR59590.1 | 88 |
| *out*B(B5S52_07150) | ARA75659.1 | AFR04340.1 | 97 | AIU89206.1 | 86 | AFI89392.1 | 82 | CAG76012.1 | 84 | AOR59589.1 | 82 |
| **Type III secretion system** | |  |  |  |  |  |  |  |  |  |  |
| *fli*I (B5S52_08465) | ARA75899.1 | AFR04086.1 | 100 | AIU88917.1 | 99 | AFI89890.1 | 99 | CAG74628.1 | 98 | AOR59124.1 | 99 |
| *fli*R (B5S52_08420) | ARA75890.1 | AFR04095.1 | 100 | AIU88926.1 | 97 | AFI89881.1 | 95 | CAG74619.1 | 96 | AOR59133.1 | 95 |
| *hrc*U (B5S52_10590) | ARA76299.1 | AFR03697.1 | 99 | AIU88652.1 | 97 | ^a^NA | ^a^NA | CAG74978.1 | 95 | ^a^NA | ^a^NA |
| *hrc*T (B5S52_10595) | ARA76300.1 | AFR03696.1 | 99 | AIU88651.1 | 96 | ^a^NA | ^a^NA | CAG74979.1 | 97 | ^a^NA | ^a^NA |
| *hrc*S (B5S52_10600) | ARA76301.1 | AFR03695.1 | 100 | AIU88650.1 | 100 | ^a^NA | ^a^NA | CAG74980.1 | 99 | ^a^NA | ^a^NA |
| *hrc*R (B5S52_10605) | ARA76302.1 | AFR03694.1 | 100 | AIU88649.1 | 99 | ^a^NA | ^a^NA | CAG74981.1 | 99 | ^a^NA | ^a^NA |
| *hrc*Q (B5S52_10610) | ARA76303.1 | AFR03693.1 | 92 | AIU88648.1 | 82 | ^a^NA | ^a^NA | CAG74982.1 | 74 | ^a^NA | ^a^NA |
| *hrp*P (B5S52_10615) | ARA76304.1 | AFR03692.1 | 99 | AIU88647.1 | 92 | ^a^NA | ^a^NA | CAG74983.1 | 82 | ^a^NA | ^a^NA |
| *hrp*O (B5S52_10620) | ARA76305.1 | AFR03691.1 | 100 | AIU88646.1 | 93 | ^a^NA | ^a^NA | CAG74984.1 | 88 | ^a^NA | ^a^NA |
| *hrc*N (B5S52_10625) | ARA76306.1 | AFR03689.1 | 99 | ^a^NA | ^a^NA | ^a^NA | ^a^NA | CAG74985.1 | 96 | ^a^NA | ^a^NA |
| *hrp*Q (B5S52_10630) | ARA76307.1 | AFR03688.1 | 99 | AIU88645.1 | 91 | ^a^NA | ^a^NA | CAG74986.1 | 90 | ^a^NA | ^a^NA |
| *hrc*V (B5S52_10635) | ARA76308.1 | AFR03687.1 | 99 | ^a^NA | ^a^NA | ^a^NA | ^a^NA | CAG74987.1 | 96 | ^a^NA | ^a^NA |
| *hrp*J (B5S52_10640) | ARA78523.1 | AFR03686.1 | 99 | AIU90497.1 | 96 | ^a^NA | ^a^NA | CAG74988.1 | 93 | ^a^NA | ^a^NA |
| *hrp*L (B5S52_10645) | ARA76309.1 | AFR03685.1 | 100 | AIU88644.1 | 97 | ^a^NA | ^a^NA | CAG74989.1 | 97 | ^a^NA | ^a^NA |
| *hrp*X (B5S52_10650) | ARA76310.1 | AFR03684.1 | 99 | AIU88643.1 | 94 | ^a^NA | ^a^NA | CAG74990.1 | 91 | ^a^NA | ^a^NA |
| *hrp*Y (B5S52_10655) | ARA76311.1 | AFR03683.1 | 99 | ^a^NA | ^a^NA | ^a^NA | ^a^NA | CAG74991.1 | 96 | ^a^NA | ^a^NA |
| *hrp*S (B5S52_10660) | ARA76312.1 | AFR03682.1 | 99 | AIU88642.1 | 92 | ^a^NA | ^a^NA | CAG74992.1 | 90 | ^a^NA | ^a^NA |
| *yla*B (B5S52_10665) | ARA76313.1 | AFR03681.1 | 99 | AIU88641.1 | 89 | AFI90588.1 | 83 | CAG74993.1 | 83 | AOR58458.1 | 83 |
| *hrp*A (B5S52_10670) | ARA76314.1 | AFR03680.1 | 100 | AIU88640.1 | 93 | ^a^NA | ^a^NA | CAG74995.1 | 93 | ^a^NA | ^a^NA |
| *hrp*B (B5S52_10675) | ARA76315.1 | AFR03679.1 | 99 | AIU88639.1 | 98 | ^a^NA | ^a^NA | CAG74996.1 | 94 | ^a^NA | ^a^NA |
| *hrc*J (B5S52_10680) | ARA76316.1 | AFR03678.1 | 99 | ^a^NA | ^a^NA | ^a^NA | ^a^NA | CAG74997.1 | 96 | ^a^NA | ^a^NA |
| *hrp*D (B5S52_10685) | ARA76317.1 | AFR03677.1 | 98 | AIU88638.1 | 87 | ^a^NA | ^a^NA | CAG74998.1 | 74 | ^a^NA | ^a^NA |
| *hrp*E (B5S52_10690) | ARA76318.1 | AFR03676.1 | 99 | ^a^NA | ^a^NA | ^a^NA | ^a^NA | CAG74999.1 | 86 | ^a^NA | ^a^NA |
| *hrp*F (B5S52_10695) | ARA76319.1 | AFR03675.1 | 100 | AIU88637.1 | 100 | ^a^NA | ^a^NA | CAG75000.1 | 99 | ^a^NA | ^a^NA |
| *hrp*G (B5S52_10700) | ARA76320.1 | AFR03674.1 | 99 | AIU88636.1 | 91 | ^a^NA | ^a^NA | CAG75001.1 | 87 | ^a^NA | ^a^NA |
| *hrc*C (B5S52_10705) | ARA76321.1 | AFR03673.1 | 99 | AIU88635.1 | 98 | ^a^NA | ^a^NA | CAG75002.1 | 95 | ^a^NA | ^a^NA |
| *hrp*T (B5S52_10710) | ARA76322.1 | AFR03672.1 | 95 | AIU88634.1 | 94 | ^a^NA | ^a^NA | CAG75003.1 | 84 | ^a^NA | ^a^NA |
| *hrp*V (B5S52_10715) | ARA76323.1 | AFR03671.1 | 98 | AIU88633.1 | 89 | ^a^NA | ^a^NA | CAG75004.1 | 78 | ^a^NA | ^a^NA |
| *hrp*N (B5S52_10720) | ARA76324.1 | AFR03670.1 | 98 | AIU88632.1 | 96 | ^a^NA | ^a^NA | CAG75005.1 | 97 | ^a^NA | ^a^NA |
| *hrp*W (B5S52_10730) | ARA76326.1 | ^a^NA | ^a^NA | AIU89461.1 | 90 | AFI88487.1 | 53 | CAG75007.1 | 22 | ^a^NA | ^a^NA |
| *dsp*E (B5S52_10775) | ARA76333.1 | AFR03665.1 | 99 | AIU88625.1 | 93 | ^a^NA | ^a^NA | CAG75015.1 | 89 | ^a^NA | ^a^NA |
| *dsp*F (B5S52_10780) | ARA76334.1 | AFR03664.1 | 99 | ^a^NA | ^a^NA | ^a^NA | ^a^NA | CAG75016.1 | 91 | ^a^NA | ^a^NA |
| *gnt*R (B5S52_10785) | ARA76335.1 | AFR03663.1 | 99 | ^a^NA | ^a^NA | AFI90586.1 | 95 | CAG75017.1 | 96 | AOR58460.1 | 95 |
| *hrp*K (B5S52_10865) | ARA76349.1 | AFR03649.1 | 99 | AIU88610.1 | 71 | ^a^NA | ^a^NA | ^a^NA | ^a^NA | ^a^NA | ^a^NA |
| **Sec-SRP secretion system** | |  |  |  |  |  |  |  |  |  |  |
| *sec*A (B5S52_03580) | ARA75015.1 | AFR04981.1 | 99 | ^a^NA | ^a^NA | AFI91974.1 | 98 | CAG76705.1 | 98 | AOR61657.1 | 98 |
| *sec*B (B5S52_21055) | ARA78220.1 | AFR01564.1 | 100 | AIU86872.1 | 99 | AFI92560.1 | 99 | CAG73091.1 | 98 | AOR61082.1 | 99 |
| *sec*D (B5S52_16410) | ARA77381.1 | AFR02457.1 | 100 | AIU87657.1 | 99 | AFI91360.1 | 98 | CAG74032.1 | 98 | AOR57725.1 | 98 |
| *sec*E (B5S52_20780) | ARA78176.1 | AFR01608.1 | 100 | AIU86912.1 | 100 | AFI88345.1 | 97 | CAG73136.1 | 99 | AOR60646.1 | 97 |
| *sec*F (B5S52_16405) | ARA77380.1 | AFR02458.1 | 100 | ^a^NA | ^a^NA | AFI91359.1 | 94 | CAG74033.1 | 94 | AOR57726.1 | 94 |
| *sec*G (B5S52_18735) | ARA77799.1 | AFR01998.1 | 100 | AIU87260.1 | 98 | AFI88830.1 | 95 | CAG73616.1 | 95 | AOR60180.1 | 95 |
| *sec*M (B5S52_03575) | ARA75014.1 | AFR04982.1 | 100 | AIU89735.1 | 95 | AFI91975.1 | 93 | CAG76706.1 | 91 | AOR61656.1 | 93 |
| *sec*Y (B5S52_02485) | ARA74820.1 | AFR05193.1 | 100 | AIU89910.1 | 100 | AFI92197.1 | 99 | CAG76908.1 | 99 | AOR61451.1 | 99 |
| *yaj*C (B5S52_16415) | ARA77382.1 | AFR02456.1 | 100 | AIU87656.1 | 100 | AFI91361.1 | 92 | CAG74031.1 | 97 | AOR57724.1 | 92 |
| *yid*C (B5S52_22260) | ARA78443.1 | AFR05610.1 | 99 | AIU90380.1 | 99 | AFI92842.1 | 96 | CAG77341.1 | 96 | AOR60839.1 | 96 |
| *fts*Y (B5S52_00555) | ARA74460.1 | AFR05514.1 | 96 | AIU90215.1 | 87 | AFI88233.1 | 83 | CAG77243.1 | 84 | AOR60746.1 | 83 |

^a^NA = not available.
