# Supplementary material for: Comparative genomic analysis of Pectobacterium carotovorum subsp. brasiliense SX309 provides novel insights into its genetic and phenotypic features
Source: BMC Genomics. 2019 Jun 13;20:486. doi: 10.1186/s12864-019-5831-x (PMC6567464; doi:10.1186/s12864-019-5831-x)
Supplement: Supplementary file 14 — Table S8. Genetic elements of T6SS-encoding gene clusters in pathogenic Pectobacterium spp. were summarized and the presence of the key T6SS structure genes is indicated for the analysed genomes. (DOCX 18 kb) [file 12864_2019_5831_MOESM14_ESM.docx]

**Table S8** Genetic elements of T6SS-encoding gene clusters in pathogenic *Pectobacterium* spp. were summarized and the presence of the key T6SS structure genes is indicated for the analysed genomes.

| Functional context | Key genes Name | Description | Accesion no. in SX309 | Accesion no. in PCC21 | Accesion no. in BC S7 | Accesion no. in SCC3193 | Accesion no. in CFBP3304 | Accesion no. in SCRI1043 | Accesion no. in RNS08.42.1A |
| --- | --- | --- | --- | --- | --- | --- | --- | --- | --- |
| Secreted substrate | *vgr*G | Type VI secretion system secreted protein VgrG | B5S52_00350  B5S52_05200  B5S52_05550  B5S52_09335  B5S52_10725 | PCC21_032250  PCC21_033020  PCC21_041400 | BCS7_14575  BCS7_16260  BCS7_16635  BCS7_19685  BCS7_20930 | W5S_0140  W5S_0359  W5S_1468  W5S_1718  W5S_1960  W5S_2409  W5S_2420  W5S_2676  W5S_3515  W5S_3590  W5S_3674  W5S_3957  W5S_4426 | A7983_02785  A7983_06790  A7983_09125  A7983_09875  A7983_13315  A7983_19560  A7983_19590  A7983_21260 | ECA2104  ECA2867  ECA3427  ECA4142  ECA4276 | A8F97_00935  A8F97_05150  A8F97_06415  A8F97_06455  A8F97_08775  A8F97_16650  A8F97_17675  A8F97_20165 |
|  | *hcp* | Type VI secretion system effector, Hcp1 family | B5S52_00355  B5S52_04220  B5S52_05420  B5S52_05450  B5S52_05545  B5S52_09330  B5S52_13980  B5S52_14140  B5S52_16680  B5S52_18295  B5S52_18460  B5S52_19450  B5S52_21035 | PCC21_032260  PCC21_041390 | BCS7_04320  BCS7_14585  BCS7_16265  BCS7_16310  BCS7_20925 | W5S_0060  W5S_0358  W5S_0553  W5S_1467  W5S_2432  W5S_2677  W5S_3516  W5S_3956  W5S_4425 | A7983_04100  A7983_07130  A7983_09130  A7983_09870  A7983_19645  A7983_21265 | ECA2866  ECA3428  ECA4275 | A8F97_05145  A8F97_06355  A8F97_16655  A8F97_20170 |
| Outer membrane protein | *vas*D | Type VI secretion system protein VasD (Putative lipoprotein) | B5S52_05495 | PCC21_032360 | BCS7_14495  BCS7_16370 | W5S_0968  W5S_2440 | A7983_12755  A7983_19685 | ECA3439 | A8F97_06315  A8F97_13560 |
| Inner membrane protein | *imp*L | Type VI secretion system protein ImpL (IcmF-related protein) | B5S52_05530 | PCC21_032290 | BCS7_04365  BCS7_14545  BCS7_16335 | W5S_0975  W5S_2437 | A7983_12790  A7983_19670 | ECA3432 | A8F97_06330  A8F97_13525 |
|  | *imp*K | Type VI secretion system protein ImpK/ DotU | B5S52_05505 | PCC21_032340 | BCS7_14595 | W5S_0970  W5S_2438 | A7983_12765  A7983_19675 | ECA3437 | A8F97_06325  A8F97_13550 |
| ATPase | *clp*V | Type VI secretion ATPase, ClpV1 family | B5S52_05510 | PCC21_032330 | BCS7_04325  BCS7_14580  BCS7_16355 | W5S_0971  W5S_2422 | A7983_12770  A7983_19600 | ECA3436 | A8F97_06405  A8F97_13545 |
| Regulatory  protein/Other structure protein | *imp*B | Type VI secretion system protein ImpB | B5S52_05465 | PCC21_032420 | BCS7_04295  BCS7_14610  BCS7_16400 | W5S_0962  W5S_2434 | A7983_12725  A7983_19655 | ECA3445 | A8F97_06345  A8F97_13590 |
|  | *imp*C | Type VI secretion system protein ImpC | B5S52_05470 | PCC21_032410 | BCS7_04300  BCS7_14605 | W5S_0963  W5S_2433 | A7983_12730  A7983_19650 | ECA3444 | A8F97_06350  A8F97_13585 |
|  | *tss*E | Type VI secretion system protein | B5S52_05475 | PCC21_032400 | BCS7_16390 | W5S_0964 | A7983_12735 | ECA3443 | A8F97_13580 |
|  | *imp*G | Type VI secretion system protein ImpG | B5S52_05480 | PCC21_032390 | BCS7_14505  BCS7_16385 | W5S_0965  W5S_2424 | A7983_12740  A7983_19610 | ECA3442 | A8F97_06395  A8F97_13575 |
|  | *imp*H | Type VI secretion system protein ImpH | B5S52_05485 | PCC21_032380 | BCS7_14500  BCS7_16380 | W5S_0966  W5S_2423 | A7983_12745  A7983_19605 | ECA3441 | A8F97_06400  A8F97_13570 |
|  | *imp*I | Type VI secretion system protein ImpI | B5S52_05490 | PCC21_032370 | BCS7_16375 | W5S_0967 | A7983_12750 | ECA3440 | A8F97_13565 |
|  | *imp*J | Type VI secretion system protein ImpJ | B5S52_05500 | PCC21_032350 | BCS7_04305  BCS7_14600  BCS7_16365 | W5S_0969  W5S_2439 | A7983_12760  A7983_19680 | ECA3438 | A8F97_06320  A8F97_13555 |
|  | *vas*H | Sigma-54 dependent transcriptional regulator | B5S52_05515 | PCC21_032320 | ^a^NA | W5S_0972 | A7983_12775 | ECA3435 | A8F97_13540 |
|  | *vas*I | Type VI secretion system protein VasI | B5S52_05520 | PCC21_032310 | BCS7_13230  BCS7_16345 | W5S_0973 | A7983_12780 | ECA3434 | A8F97_13535 |
|  | *vas*J | Type VI secretion system protein VasJ | B5S52_05525 | PCC21_032300 | BCS7_14540  BCS7_16340 | W5S_0974 | A7983_12785 | ECA3433 | A8F97_13530 |
|  | *vas*L | Type VI secretion system protein VasL | B5S52_05535 | PCC21_032280 | BCS7_14475  BCS7_16330 | W5S_0976  W5S_2435 | A7983_12795  A7983_19660 | ECA3431 | A8F97_06340  A8F97_13520 |
|  | *tss*I | Type VI secretion protein TssI | ^a^NA | ^a^NA | ^a^NA | W5S_2410 | A7983_19565 | ^a^NA | A8F97_06450 |
|  | *imp*F | Type VI secretion system lysozyme like protein | ^a^NA | ^a^NA | ^a^NA | W5S_2425 | A7983_19615 | ^a^NA | A8F97_06390 |
|  | *imp*E | Virulence protein SciE type | ^a^NA | ^a^NA | ^a^NA | W5S_2426 | A7983_19620 | ^a^NA | A8F97_06385 |
|  | *imp*M | Type VI secretion system protein Imp | ^a^NA | ^a^NA | ^a^NA | W5S_2436 | A7983_19665 | ^a^NA | A8F97_06335 |

^a^NA = not available.
