# Supplementary material for: Comparative genomic analysis of Pectobacterium carotovorum subsp. brasiliense SX309 provides novel insights into its genetic and phenotypic features
Source: BMC Genomics. 2019 Jun 13;20:486. doi: 10.1186/s12864-019-5831-x (PMC6567464; doi:10.1186/s12864-019-5831-x)
Supplement: Supplementary file 16 — Table S9. Identification of homologs of quorum sensing genes in P. carotovorum subsp. brasiliense SX309 and other Pectobacterium spp. (DOCX 16 kb) [file 12864_2019_5831_MOESM16_ESM.docx]

**Table S9** Identification of homologs of quorum sensing genes in *P. carotovorum* subsp. *brasiliense* SX309 and other *Pectobacterium* spp.

| **Genes in SX309^*^** | **Accesion** | **PCC21** | | **BC S7** | | **SCC3193** | | **SCRI1043** | | **RNS08.42.1A** | |
| --- | --- | --- | --- | --- | --- | --- | --- | --- | --- | --- | --- |
|  | **no. in SX309** | **Accesion** | **Homology** | **Accesion** | **Homology** | **Accesion** | **Homology** | **Accesion** | **Homology** | **Accesion** | **Homology** |
|  |  | **no.** | **(%)** | **no.** | **(%)** | **no.** | **(%)** | **no.** | **(%)** | **no.** | **(%)** |
| **AHLs (N-Acyl homoserine lactones) quorum-sensing system** | | | |  |  |  |  |  |  |  |  |
| *car*I (B5S52_21425) | ARA78288.1 | AFR01497.1 | 100 | AIU86811.1 | 99 | AFI92653.1 | 74 | CAG73025.1 | 98 | AOR61019.1 | 97 |
| *exp*R (B5S52_21420) | ARA78287.1 | AFR01498.1 | 100 | AIU86812.1 | 99 | AFI92652.1 | 62 | CAG73026.1 | 95 | AOR61020.1 | 93 |
| **LuxS/AI-2 (Autoinducer 2) quorum-sensing system** | | | |  |  |  |  |  |  |  |  |
| *lux*S (B5S52_05735) | ARA75404.1 | AFR04598.1 | 99 | AIU89425.1 | 100 | AFI89133.1 | 97 | CAG76260.1 | 98 | AOR59868.1 | 97 |
| *rbs*B (B5S52_21960) | ARA78384.1 | AFR05664.1 | 100 | AIU90349.1 | 100 | AFI92752.1 | 100 | CAG72937.1 | 99 | AOR60927.1 | 100 |
| **QS based on two-component regulators** | | | |  |  |  |  |  |  |  |  |
| *qse*B (B5S52_21985) | ARA78389.1 | AFR05659.1 | 99 | AIU90344.1 | 97 | AFI92757.1 | 95 | CAG72932.1 | 96 | AOR60922.1 | 95 |
| *qse*C (B5S52_21990) | ARA78390.1 | AFR05658.1 | 99 | AIU90343.1 | 94 | AFI92758.1 | 91 | CAG72931.1 | 92 | AOR60921.1 | 92 |
| *cpx*A (B5S52_00755) | ARA74496.1 | AFR05479.1 | 100 | AIU90172.1 | 99 | AFI88268.1 | 98 | CAG77209.1 | 99 | AOR60712.1 | 98 |
| *cpx*R (B5S52_00760) | ARA74497.1 | AFR05478.1 | 100 | AIU90173.1 | 99 | AFI88269.1 | 98 | CAG77208.1 | 99 | AOR60711.1 | 98 |
| *cpx*P (B5S52_00765) | ARA74498.1 | AFR05477.1 | 99 | AIU90174.1 | 92 | AFI88270.1 | 90 | CAG77207.1 | 93 | AOR60710.1 | 89 |
| *gac*S (B5S52_04715) | ARA75230.1 | AFR04798.1 | 99 | AIU89584.1 | 96 | AFI91750.1 | 95 | CAG76469.1 | 95 | AOR57471.1 | 95 |
| *gac*A (B5S52_08195) | ARA75851.1 | AFR04138.1 | 100 | AIU89005.1 | 100 | AFI89552.1 | 99 | CAG75782.1 | 99 | AOR59321.1 | 99 |
